# Integration of Multiple Platforms for the Analysis of Multifluorescent Marking Technology Applied to Pediatric GBM and DIPG

**DOI:** 10.3390/ijms21186763

**Published:** 2020-09-15

**Authors:** Giulia Pericoli, Stefania Petrini, Ezio Giorda, Roberta Ferretti, Maria Antonietta Ajmone-Cat, Will Court, Libenzio Adrian Conti, Roberta De Simone, Paola Bencivenga, Alessia Palma, Angela Di Giannatale, Chris Jones, Andrea Carai, Angela Mastronuzzi, Emmanuel de Billy, Franco Locatelli, Maria Vinci

**Affiliations:** 1 Department of Onco-Hematology, Cell and Gene Therapy, Bambino Gesù Children’s Hospital—IRCCS, 00146 Rome, Italy; giulia.pericoli@opbg.net (G.P.); roberta.ferretti@opbg.net (R.F.); angela.digiannatale@opbg.net (A.D.G.); angela.mastronuzzi@opbg.net (A.M.); emmanuel.decrespin@opbg.net (E.d.B.); franco.locatelli@opbg.net (F.L.); 2 Confocal Microscopy Core Facility, Research Center, Bambino Gesù Children’s Hospital—IRCCS, 00146 Rome, Italy; stefania.petrini@opbg.net (S.P.); libenzioadrian.conti@opbg.net (L.A.C.); 3 FACs Core Facility, Bambino Gesù Children’s Hospital—IRCCS, 00146 Rome, Italy; ezio.giorda@opbg.net; 4 National Centre for Drug Research and Evaluation, Istituto Superiore di Sanità, 00161 Rome, Italy; mariaantonietta.ajmone-cat@iss.it (M.A.A.-C.); roberta.desimone@iss.it (R.D.S.); 5 Cancer Research UK Cancer Therapeutics Unit, The Institute of Cancer Research, Sutton SM2 5NG, UK; will.court@icr.ac.uk; 6 Research Laboratories, Bambino Gesù Children’s Hospital—IRCCS, 00146 Rome, Italy; paola.bencivenga@opbg.net (P.B.); alessia.palma@opbg.net (A.P.); 7 Division of Molecular Pathology, Institute of Cancer Research, Sutton SM2 5NG, UK; chris.jones@icr.ac.uk; 8 Department of Neuroscience and Neurorehabilitation, Bambino Gesù Children’s Hospital—IRCCS, 00165 Rome, Italy; andrea.carai@opbg.net

**Keywords:** pediatric GBM, DIPG, multifluorescent marking technology, RGB marking, optical barcode, fluorescence imaging, heterogeneity

## Abstract

The intratumor heterogeneity represents one of the most difficult challenges for the development of effective therapies to treat pediatric glioblastoma (pGBM) and diffuse intrinsic pontine glioma (DIPG). These brain tumors are composed of heterogeneous cell subpopulations that coexist and cooperate to build a functional network responsible for their aggressive phenotype. Understanding the cellular and molecular mechanisms sustaining such network will be crucial for the identification of new therapeutic strategies. To study more in-depth these mechanisms, we sought to apply the Multifluorescent Marking Technology. We generated multifluorescent pGBM and DIPG bulk cell lines randomly expressing six different fluorescent proteins and from which we derived stable optical barcoded single cell-derived clones. In this study, we focused on the application of the Multifluorescent Marking Technology in 2D and 3D in vitro/ex vivo culture systems. We discuss how we integrated different multimodal fluorescence analysis platforms, identifying their strengths and limitations, to establish the tools that will enable further studies on the intratumor heterogeneity and interclonal interactions in pGBM and DIPG.

## 1. Introduction

Pediatric Glioblastoma (pGBM) and Diffuse Intrinsic Pontine Glioma (DIPG), are amongst the most aggressive tumors of the central nervous system affecting children and young adults, for which there is no effective treatment [1,2]. Integrated molecular profiling has revealed that these neoplasms are characterized by recurrent specific mutations in histone genes together with aberrations in canonical oncogenic pathways associated with differences in tumor location, histopathological features, patient age distribution, and clinical outcome [3,4,5,6,7]. The histone mutations involve the H3.3 (*H3F3A*) and H3.1 (*HIST1H3B, HIST1H3C*) variants, resulting in amino-acid substitutions (G34R/V and K27M) at the histone tail interfering with their natural function.

A significant degree of genetic and phenotypic intratumor heterogeneity has been recently identified in pGBM and DIPG, which may represent one of the most challenging aspect in the effort to develop new effective therapeutic strategies for these diseases [8,9,10,11,12]. It has been shown that they are characterized by temporal and spatial intratumoral genomic heterogeneity, which rapidly evolves following surgical and chemotherapy treatment. Also, it has been recently demonstrated that pGBMs and DIPGs are characterized by a complex and heterogenous sub-clonal architecture, where distinct cell subpopulations coexist and co-operate, building a cellular network promoting tumorigenesis and responsible of the aggressive phenotype [12]. Moreover, using single-cell RNA sequencing, it has been shown that these cells exhibit high plasticity in the transition between different cellular states and that it can be influenced by the microenvironment [13]. Of note, evidence has clearly demonstrated the existence of direct interconnections between the neuronal compartment of the brain microenvironment wiring the glioma cells and vice versa [14,15].

Despite the increasing knowledge, questions remain to be answered. For example, what are the precise mechanisms that regulate the direct or indirect cell–cell communication within the heterogeneous glioma populations and with its microenvironment? How can we interfere with the mechanisms of crosstalk in order to weaken glioma growth and invasion? How do the heterogeneous subpopulations evolve over time upon therapeutic selective pressure?

In order to be able to study these mechanisms at cellular and molecular levels, we would need to identify and track individual cells and/or specific cell subpopulations.

The RGB marking technology [16] was originally developed for the simultaneous cell transduction of three lentiviral gene ontology (LeGO) vectors, encoding for red-green-blue fluorescent proteins. This technology has recently evolved with the use of up to six multiple vectors, each expressing fluorescent proteins with distinct excitation and emission properties, allowing the generation of multifluorescent cell populations, stable through cell divisions [17]. The RGB marking approach has been used to assess the clonality of primary hepatocytes in the regeneration of injured livers in mice, to track the spatial and temporal fate of neural stem cells in the adult brain as well as to study tumor heterogeneity in terms of clonal expansion in vitro and in vivo [16,17,18].

In this study we have applied the Multifluorescent Marking Technology adapted from the RGB marking approach [16,17], to pGBM and DIPG patient primary derived cell lines. We describe the generation of multifluorescent bulk cell lines, the derivation of stable optical barcoded single cell-derived clones and the assessment of the Multifluorescent Marking Technology in 2D and 3D in vitro/ex vivo culture systems. We focus on how we can integrate different fluorescence analysis platforms and identify their strengths and limitations, with the aim to establish the tools that will enable future more in-depth studies on the crosstalk between distinct heterogeneous subpopulations in pGBM and DIPG.

## 2. Results

### 2.1. Generation of Multifluorescent pGBM and DIPG Cell Lines

**Figure 1:**
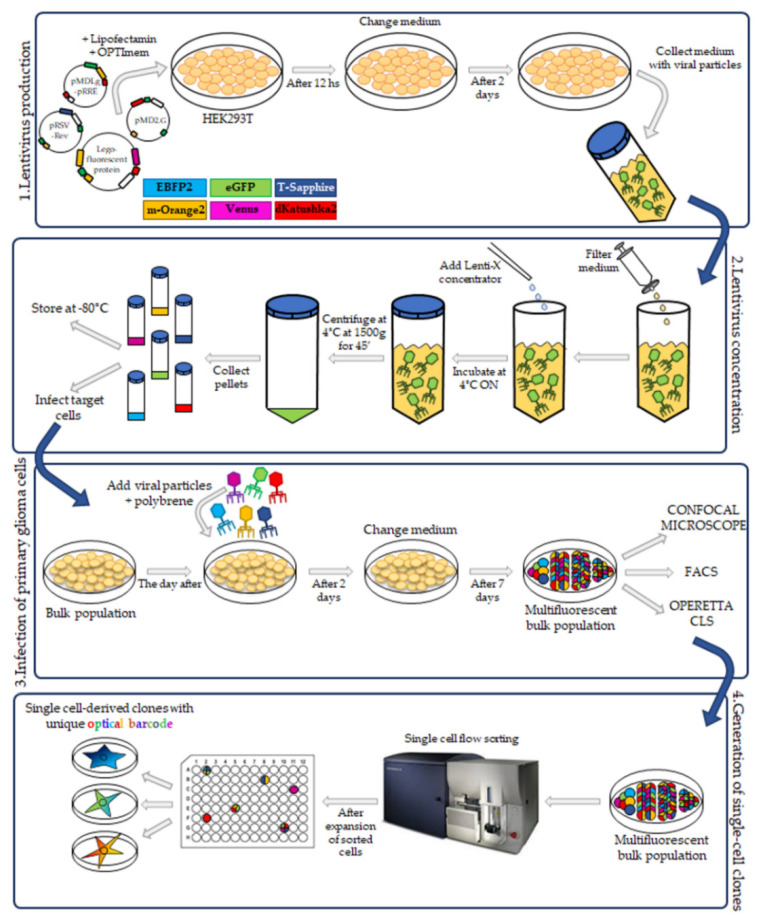
Workflow— pediatric glioblastoma (pGBM) and diffuse intrinsic pontine glioma (DIPG) cell transduction for the generation of multifluorescent bulk cell lines and optical barcoded single cell-derived clones. Schematic workflow from the lentivirus production of six different lentiviral gene ontology (LeGO) vectors for the Multifluorescent Marking Technology. Lentivirus vectors were produced thanks to the transient transfection of HEK-293T cells. The medium was collected, and the viral particles were concentrated and subsequently used for the pGBM and DIPG primary cell lines infection to generate the multifluorescent bulk primary cell lines. The primary multifluorescent bulk population was single cell-flow sorted into 96-well plates to grow and establish single cell-derived clones characterized by specific optical barcodes. Multifluorescent bulk population and derived clones were characterized for their fluorescent make up by using different multimodal platforms: Flow Cytometer, Confocal microscope, and Operetta CLS.

We applied the Multifluorescent Marking Technology to our pGBM and DIPG patient primary derived cell lines. For this, we used the Lentiviral Gene Ontology (LeGO) vectors, previously described for the RGB marking and Optical Barcoding [17,19], expressing six different fluorescent proteins with distinct excitation and emission properties: LeGO-G2 (eGFP), LeGO-V2 (Venus), LeGO-S2 (T-Sapphire), LeGO-mOrange2 (m-Orange2), LeGO-EBFP2 (EBFP2) and LeGO-dKatushka2 (dKatushka2) (Figure 1, 1. Lentivirus production).

To transduce pGBM and DIPG primary cell lines, we followed the workflow as reported in Figure 1. We used the RGB marking procedure previously reported [19] with some modifications. As our patient primary derived cell lines are exclusively grown in stem cell-like culture condition [12], the original protocol was modified in order to remove the serum from the viral preparation. Briefly, to produce the lentiviral particles, the HEK293T cells were transiently transfected with each of the six LeGO vectors separately, in the presence of the packaging plasmids (Figure 1, 1. Lentivirus production). After 60 h, the medium was collected, and the virus purified and concentrated using the Lenti-X concentrator to remove from the HEK293T viral-medium the serum (FBS) and potential cell debris. Then, the viral pellet was resuspended in phosphate-buffered saline (PSB) and diluted in the Stem Cell Medium (Figure 1, 2. Lentivirus concentration).

The pGBM and DIPG primary cell lines, normally expanded in 3D as neurospheres, were cultured adherent on laminin [20] to ensure a higher cell transduction efficiency and to facilitate the visualization of the transduced fluorescent proteins under a microscope.

The cells were transduced with equal amounts of all six lentiviral vectors simultaneously at a Multiplicity of Infection (MOI) of 0.7, corresponding to a transduction rate of 50% for each lentivirus (Figure 1, 3. Infection of primary glioma cells). Since our cell lines are primary cultures, generally more difficult to transduce [19] than established cell lines, we tested the amount of viral particles required to obtain the optimal condition of cell transduction. We found that the viral particles used 100-fold concentrated respect to the original titer, gave good transduction efficiency.

Upon infection, the expression of the fluorescent proteins was monitored using a Leica DMi8 fluorescence microscope and, after cell expansion, the multifluorescent pGBM and DIPG cell lines were analyzed on three different fluorescence platforms: BD FacsAria^TM^ III flow cytometer, Leica TCS AOBS-SP8X confocal microscope and Operetta CLS.

In order to generate single cell-derived clones (Figure 1, 4. Generation of single-cell clones) the multifluorescent bulk cell populations were single cell-flow sorted, and individual colonies expanded. The single cell-derived clones were subsequently analyzed to determine the specific optical barcode (OB) given by the combination of the individual fluorescent proteins expressed.

### 2.2. Multimodal Analysis of Multifluorescent Marking Technology

The Multifluorescent Marking Technology was performed on four pHGG patient derived cell lines, representing different locational and mutational subgroups (Table 1 and Figure S1): two DIPG cell lines, OPBG-DIPG002 (pons H3.3 K27M) and OPBG-DIPG004 (pons H3.1 K27M) and two pGBM primary cell lines, OPBG-GBM002 (hemispheric histone WT) and OPBG-GBM001 (hemispheric H3.3 G34R). Three days after infection, we checked the transduction efficiency on an inverted fluorescence microscope. Although this microscope is limited only to the detection of red, green, and blue fluorescences, it gave us at this initial stage, the possibility to check the cell transduction, which reached a maximum intensity about a week after infection.

#### 2.2.1. Flow Cytometry and FACS Analysis

**Figure 2:**
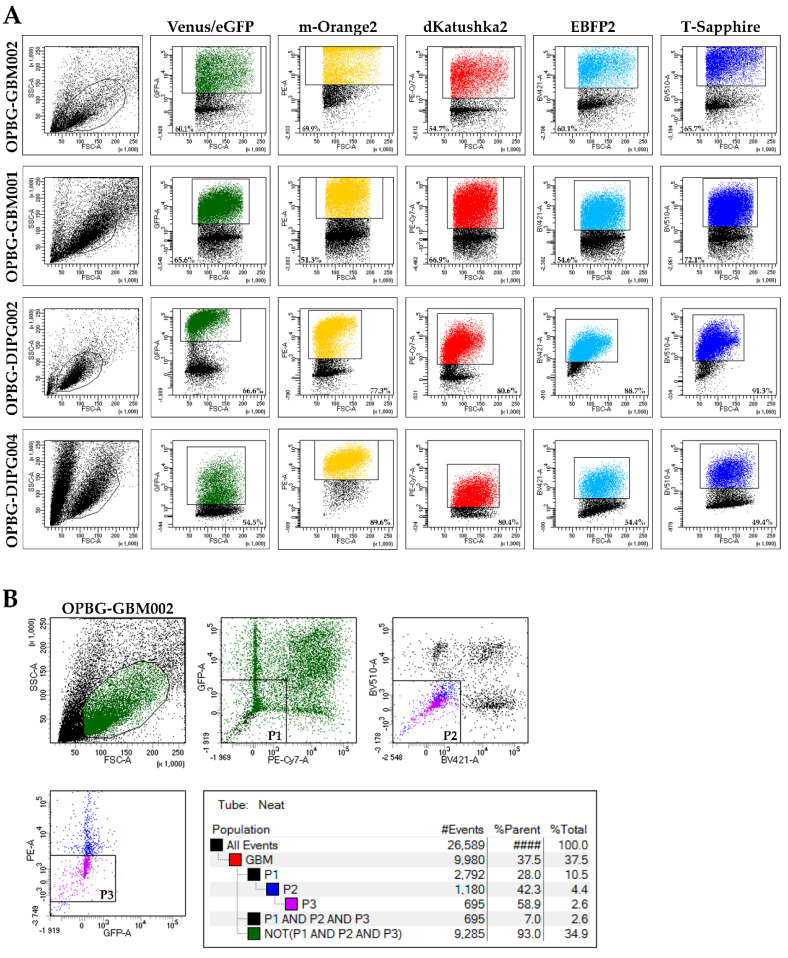
Flow cytometry and FACS analysis of the pGBM and DIPG multifluorescent primary cell lines. (**A**) The pGBM and DIPG multifluorescent cell lines flow cytometry analysis shows the differential transduction efficiency of four instead of six fluorescent LeGO vectors. The six different fluorescences were analyzed with different emission detection range GFP-530/30 (eGFP), GFP-545/35 (Venus), PE-582/15 (m-Orange2), PE-Cy7-780/60 (dKatushka2), BV421-450/40 (EBFP2) and BV510-510/50 (T-Sapphire). (**B**) FACS gating strategy example used to perform the single cell-flow sorting of pGBM and DIPG multifluorescent bulk population. The FACS analysis was performed using a flow cytometer with cell-sorting capability (BD FacsAria^TM^ III). The exemplified experiment is relative to OPBG-GBM002 multifluorescent bulk cell line.

For each patient-derived cell line, the transduction efficiency for the individual lentiviral vector was verified by flow cytometry analysis in order to determine the percentage of cells positive to each fluorescent protein in the bulk cell population (Figure 2). The filter configuration of our flow cytometer (Table 2) enabled us to discriminate only four out of six fluorescence markers. Given the close range of emission wavelengths, we could successfully separate the m-Orange2 from dKatushka2, and the EBFP2 from T-Sapphire. However, we could not distinguish the Venus from eGFP due to a strong overlap between the two emission spectra. Therefore, the analysis relative to the transduction efficiency could only be performed for four out of the six fluorescent proteins, excluding Venus and eGFP (Figure 2A).

For the GBM cell lines, OPBG-GBM001 and OPBG-GBM002, all four LeGO vectors gave similar transfection efficiencies between 51–72% and 54–70% respectively, with high fluorescent intensity range (10^3^–10^5^) (Figure 2A). Although we could not separate Venus from eGFP, we had 65.6% of OPBG-GBM001 and 60.1% of OPBG-GBM002 cells detected in the emission range corresponding to both fluorescences.

With the DIPG cell lines we had different results. The OPBG-DIPG002 showed a higher efficiency of transduction for the four lentiviral vectors between 67–91% and the fluorescence intensity for the four detected proteins was variable (Figure 2A). In particular, m-Orange2, dKatushka2, and T-Sapphire had fluorescence intensity ranging from 10^3^ to 10^5^, while EBFP2 showed a lower fluorescent intensity from 10^3^ to 10^4^. In contrast, the OPBG-DIPG004 showed a lower transduction efficiency for the four vectors, between 49–89% and a wider range of fluorescence intensity from 10^2^ to 10^5^ (Figure 2A). For the two proteins with the overlapping spectra, Venus and eGFP, we had 66.6% of OPBG-DIPG002 and 54.5% of OPBG-DIPG004 positive cells.

In order to apply the Multifluorescent Marking Technology to the study of intratumor heterogeneity in pGBM and DIPG patient primary derived cell lines, we generated single cell-derived clones upon single cell-flow sorting in 96-well plates using the BD FacsAria^TM^ III flow cytometer. We adopted a specific sorting strategy aimed to retrieve only the cells positive for any (one or more) of the six fluorescent proteins (Figure 2B). To achieve this, we performed gating in order to exclude the non-fluorescent cells for the analyzed fluorescences (P1: eGFP/Venus–dKatushka2, P2: T-Sapphire–EBFP2 and P3: eGFP/Venus–m-Orange2) and the intersection of these three gates (P1 and P2 and P3). Then, the depletion of this intersected pool defined as “NOT (P1 and P2 and P3)”, allowed us to successfully sort only the fluorescent cells.

#### 2.2.2. Confocal Microscope

Following the cell sorting, the multifluorescent bulk cell lines were expanded, and between passages 12–15, were characterized for their acquired multifluorescent protein expression at a Leica TCS AOBS-SP8X confocal microscope. This confocal microscopy platform is equipped with a tunable pulsed single diode white light-laser (WLL, tuning range of 470–670 nm in 1nm intervals) and a tunable acousto-optical beam splitter (AOBS). This allowed us to selectively set the excitation laser line and the emission range by blocking sliders in front of the detectors (Table 3 and Figure S2) that ensured the finest separation and visualization of the six different fluorescent proteins for each cell line.

**Figure 3:**
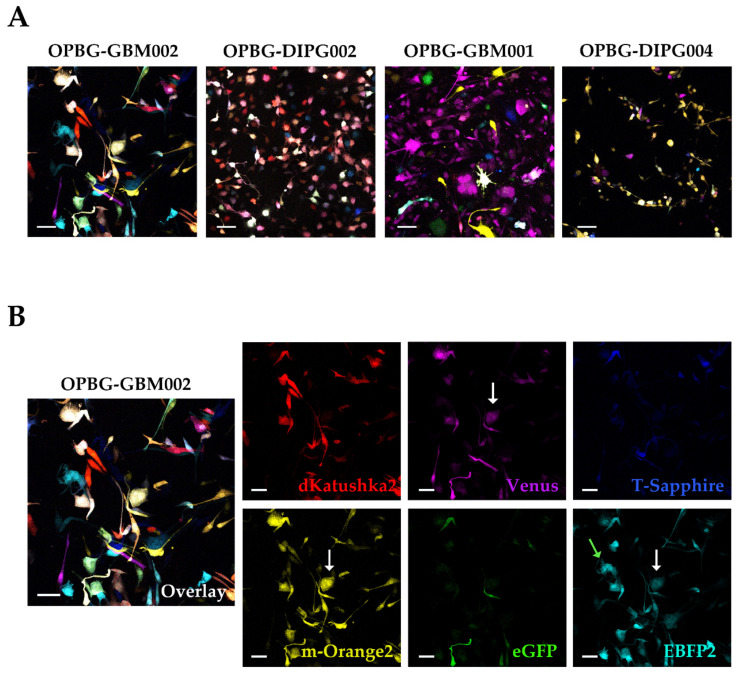
Analysis at the confocal microscope of pGBM and DIPG multifluorescent primary cell lines. (**A**) OPBG-GBM002, OPBG-DIPG002, OPBG-GBM001, and OPBG-DIPG004 patient primary cell lines were transduced with six different fluorescent LeGO vectors. Representative merged fluorescent images are shown for each cell line. (**B**) The representative merged fluorescent image for OPBG-GBM002, is splitted into six panels, one for each protein: dKatushka2 (red), m-Orange2 (yellow), Venus (magenta), eGFP (green), T-Sapphire (blue), and EBFP2 (cyan). Light green and white arrows indicate respectively one-fluorescence and multi-fluorescence cells. All images were acquired using a confocal microscope (Leica TCS AOBS-SP8X). Scale bars: 10 µm.

The representative images relative to the four multifluorescent cell lines clearly demonstrated the co-expression of multiple fluorescences, appearing as different rainbow-like images (Figure 3A). Given that cells are randomly infected by one or more lentiviral vector and can integrate different copies of each vector, we could identify cells that expressed one (Figure 3B light green arrow) or more fluorescences (Figure 3B white arrows) with different intensity in the multifluorescent bulk cell lines. This is well exemplified in Figure 3B with the OPBG-GBM002 cell line. This variegated fluorescence was also observed for the other three cell lines (Figure S3A–C).

The four primary cell lines appeared to have overall a different and random uptake of the combination of the six LeGO vectors. A more uniform multicolor effect was observed in the OPBG-GBM002 and OPBG-DIPG002 cell lines compared to OPBG-GBM001 and OPBG-DIPG004, where Venus and m-Orange2 appeared respectively more dominant.

#### 2.2.3. Operetta CLS

Then, we characterized the multifluorescence of two of our bulk cell lines (OPBG-GBM002 and OPBG-DIPG002) using the Operetta CLS. This is an imaging platform for high-content/high-throughput image acquisition and analysis. It can acquire images in brightfield, digital phase and fluorescence, and being a spinning-disk confocal platform, it can be used in confocal mode for the acquisition of fluorescent images in Z-stack. The Operetta CLS configuration at our disposal is equipped with eight LED light sources with up to eight excitation wavelength ranges and eight different emission filters (Table 4) but, despite the wide range of wavelengths, we were not able to discriminate all the fluorescences. We could separate dKatushka2 from m-Orange2, but as for the flow cytometer, we were not able to discriminate Venus and eGFP or T-Sapphire and EBFP2 because of the overlap of their emission spectra.

**Figure 4:**
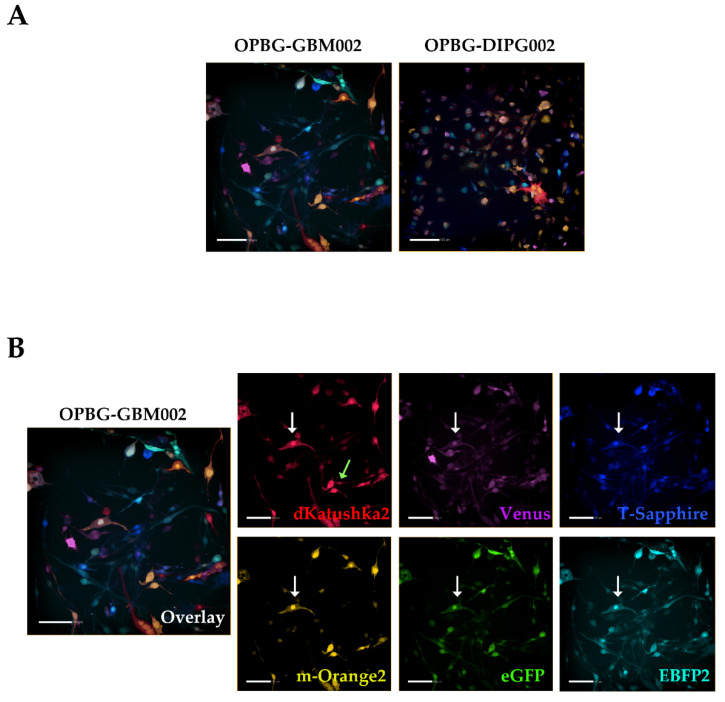
pGBM and DIPG multifluorescent primary cell lines analyzed at the Operetta CLS. (**A**) Representative images of the OPBG-GBM002 and OPBG-DIPG002 multifluorescent bulk cell lines, acquired on Operetta CLS. (**B**) The representative merged fluorescent image for OPBG-GBM002 is splitted into six panels, one for each protein: dKatushka2 (red), m-Orange2 (yellow), Venus (magenta), eGFP (green), T-Sapphire (blue), and EBFP2 (cyan). Light green and white arrows indicate respectively one-fluorescence and multifluorescent cells. All images were acquired using Operetta CLS fully equipped with 8 different emission filters (see also Table 4). Scale bars: 100 µm.

Representative images of the multifluorescent OPBG-GBM002 and OPBG-DIPG002 bulk cell lines are shown in Figure 4A.

As for the confocal, with the Operetta CLS we were able to identify cells that expressed multiple fluorescences with different intensities (Figure 4B white arrows) and cells that show only one fluorescence, exemplified with the images of the OPBG-GBM002 cell line (Figure 4B, light green arrow). However, for the multifluorescent OPBG-DIPG002 cell line, we were unable to identify cells that expressed only one fluorescence. We observed that all transduced cells were positive for at least two fluorescent proteins. This is likely due to the overlap of the excitation/emission spectra and to the filter set-up of the instrument as detailed above (Figure S4).

### 2.3. Single Cell-Derived Clones

**Figure 5:**
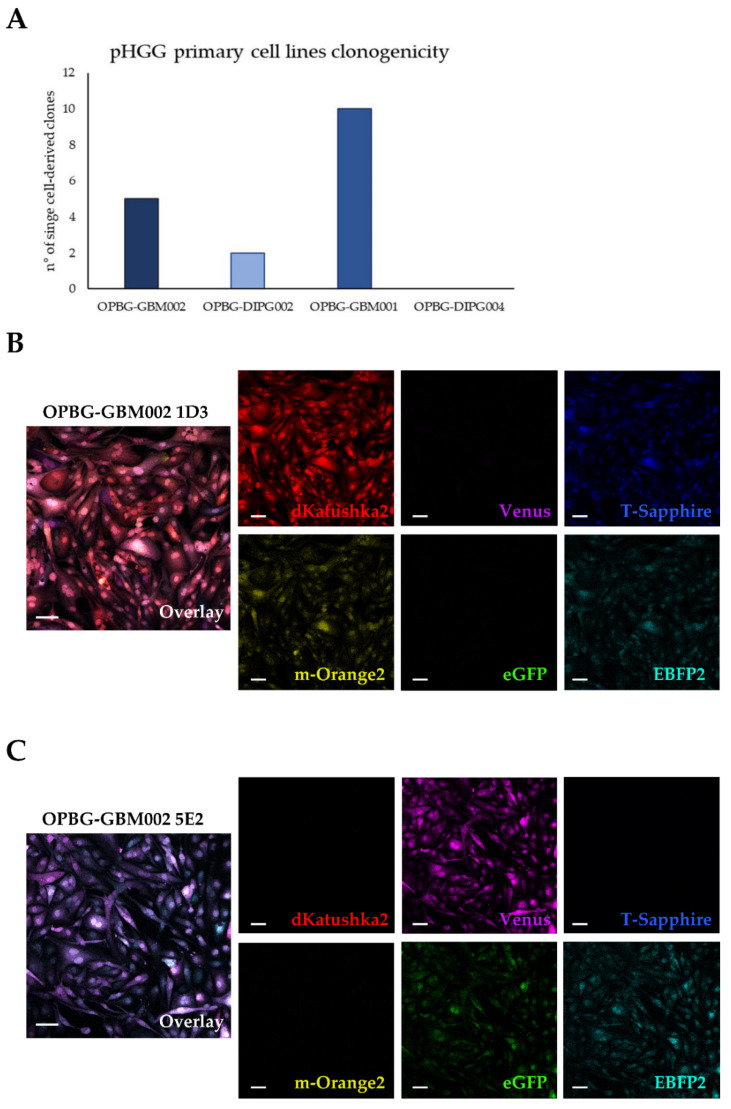
pGBM and DIPG optical barcoded single cell-derived clones analyzed on the confocal microscope. (**A**) Clonogenicity. The graph shows the number of single cells from which a cell line was established, under 2D laminin-adherent stem cell culture conditions, for the four multifluorescent primary patient-derived cell lines. (**B**,**C**) Representative fluorescent images from two OPBG-GBM002 single cell-derived clones are shown with the overlay and the splits for the six fluorescent proteins, which clearly identify the corresponding OBs for each clone: 1D3 (**B**) expressing dKatushka2 (red), m-Orange2 (yellow), T-Sapphire (blue), and EBFP2 (cyan); 5E2 (**C**) expressingVenus (magenta), eGFP (green) and EBFP2 (cyan). Fluorescent image acquisition was performed using a Leica TCS AOBS-SP8X confocal microscope. Scale bars: 10 µm.

To investigate several aspects of pGBM and DIPG intratumor heterogeneity, we generated single cell-derived colonies from the multifluorescent bulk cell lines. (Table 1). As described above, using the BD FacsAria^TM^ III flow cytometer, and our cell-sorting strategy, the multifluorescent bulk populations were single cell-flow sorted in 96-well plates pre-coated with laminin (Figure 2B). The four cell lines were observed to be heterogeneously clonogenic (Figure 5A) giving rise to different numbers of single cell-derived colonies, from which stable clones were established.

In particular, from OPBG-GBM001 and OPBG-GBM002 we established 10 and 5 single cell-derived clones respectively, and 2 clones were derived from the OPBG-DIPG002. From the OPBG-DIPG004 we did not obtain colonies. The single cell-derived clones were analyzed with the confocal microscope for their fluorescent make-up in order to determine and assign a specific OB to each clone. As shown for the OPBG-GBM002, the clone 1D3 expressed dKatushka2, m-Orange2, T-Sapphire and EBFP2, each of them with different expression levels, but was negative for Venus and eGFP (Figure 5B); the clone 5E2 expressed Venus, eGFP and EBFP2, while it did not express dKatushka2, m-Orange2 and T-Sapphire (Figure 5C). For the two clones derived from the OPBG-DIPG002 multifluorescent bulk cell line, the clone 1C5 expressed m-Orange2, Venus and dKatushka2, the latter at very low expression level (Figure S5A); the clone 2B4 expressed m-Orange2 and dKatushka2 (Figure S5B). Therefore, the Multifluorescent Marking Technology allowed the generation single-cell-derived clones, which could be assigned specific OBs, making easily distinguishable cell clones one to each other.

**Figure 6:**
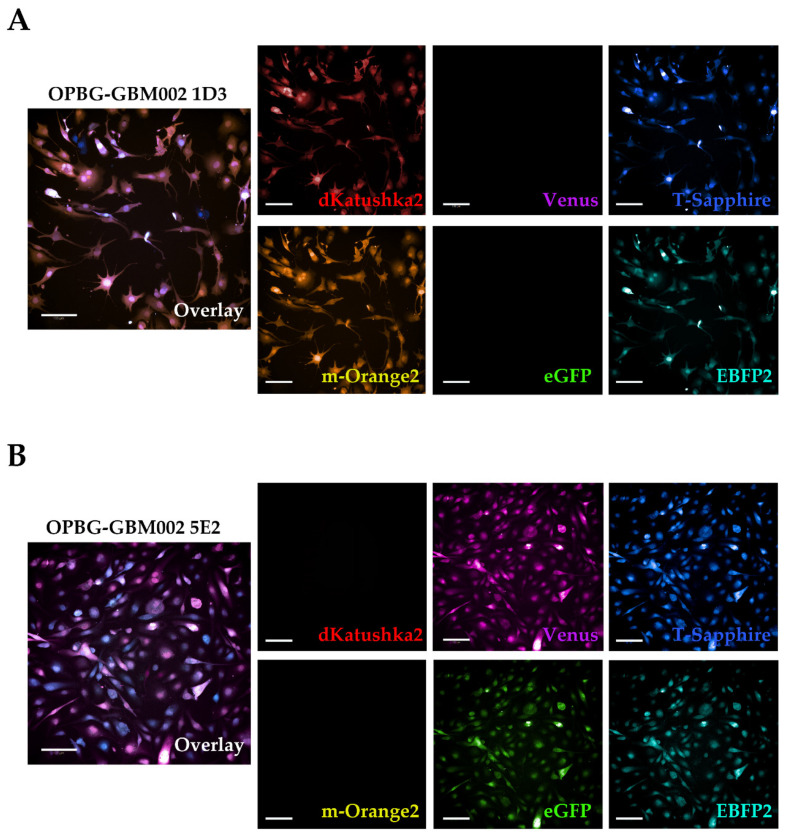
pGBM and DIPG optical barcoded single cell-derived clones analyzed on the Operetta CLS. (**A**,**B**) Representative fluorescent images of two OPBG-GBM002 single cell-derived clones, are shown. The image overlay of the combined fluorescence for the clones, are shown together with the split for the six fluorescent proteins. The clone 1D3 (**A**) appeared positive to dKatushka2 (red), m-Orange2 (yellow), T-Sapphire (blue) and EBFP2 (cyan), and the clone 5E2 (**B**) appeared positive to Venus (magenta), eGFP (green), T-Sapphire (blue), and EBFP2 (cyan). All images were acquired using Operetta CLS fully equipped with 8 different emission filters. Scale bars: 100 µm.

As expected, we could not fully distinguish all the fluorescent markers using the Operetta CLS, making it difficult to associate a unique OBs to each clone. For the clones derived from OPBG-GBM002, relatively to 1D3, we could detect dKatushka2, m-Orange2, T-Sapphire, and EBFP2, which were the same fluorescences also detected at confocal microscope (Figure 6A). For the clone 5E2, we detected Venus, eGFP, EBFP2 and T-Sapphire (Figure 6B) and unlike the OB obtained on the confocal microscope, on the Operetta CLS, a fluorescence signal corresponding to the emission spectra of T-Sapphire was also detected. We also examined OPBG-DIPG002 single cell-derived clones, and for the clone 1C5, we detected dKatushka2, m-Orange2, Venus and eGFP (Figure S6A), while for 2B4, dKatushka2 and m-Orange2 were detected (Figure S6B). As discussed above, due to the spectra overlap of some of the fluorescent proteins and the current filter set up on the Operetta CLS, the clone OBs could not be determined as precisely as on the confocal microscope.

In addition, we analyzed the fluorescence intensity in 2 bulk cell lines and 2 representative clones, one for each cell line (Figure S7). The fluorescence intensity was analyzed as the mean and per single cell, on images acquired at both the TSC SP8 confocal microscope and the Operetta CLS. Quantitative differences emerge between the two platforms due to the different resolution between optics being applied and the different width of the wavelength range that has been captured for each emission fluorescence. However, and as expected, the clones show a more uniform expression of the fluorescence intensity (Figure S7F,H), compared to the bulk cell lines from which they were derived (Figure S7B,D).

### 2.4. 3D Tumor Models

Next, we exploited the application of the Multifluorescent Marking Technology in two different DIPG 3D invasion models.

Using the Multifluorescent Marking Technology in a 3D environment would enable us to study the intratumor heterogeneity in more complex environments than in a 2D culture system and to investigate at longer term, compared to transient cell labeling [12], the direct interactions between heterogeneous cell populations.

#### 2.4.1. In vitro 3D Tumor Invasion Assay in Matrigel

**Figure 7:**
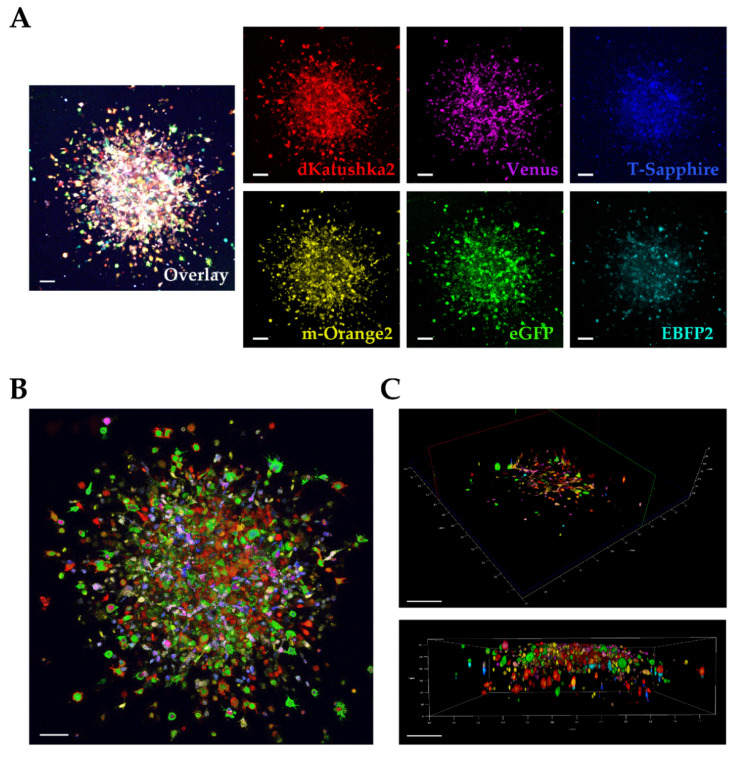
Multifluorescent in vitro 3D tumor invasion into Matrigel. (**A**) Maximum intensity projection (MIP) of z-series and complete mosaic image of multifluorescent OPBG-DIPG002 cell invasion is shown. The fluorescences of invading cells are shown in red (dKatushka2), yellow (m-Orange2), magenta (Venus), green (eGFP), blue (T-Sapphire), and cyan (EBFP2). Sequential confocal images were acquired using 10× or 20× objectives (Leica Microsystems) with a 1024 × 1024 format, and z-step size of 1 µm. Scale bar: 100 µm. Images are relative to end point invasion assay (96 h). (**B**,**C**) Deconvolution analysis (HyVolution2 software, Huygens) was applied to z-stacks to improve contrast and resolution of confocal raw images, then deconvolved images were imported into LASX 3D (Leica Microsystems) software to obtain their surface 3D rendering. Scale bars: 100 µm in (**B**), 200 µm in (**C**).

We performed an in vitro 3D tumor invasion assay [12,21,22] for which we used the OPBG-DIPG002 multifluorescent bulk cell line. The DIPG cells were grown as neurospheres up to a diameter of approximately 300–350 μm, then embedded into Matrigel for 96 h to let the cells to invade the matrix. After that, the invasions were fixed and the fluorescences analyzed at the confocal microscope. In particular, the images were acquired in z-stack with a z-step size of 1 µm. By doing so, we could successfully distinguish every cell from each other and separate the individual fluorescences in this complex in vitro 3D microenvironment (Figure 7A). To improve the contrast and resolution of the confocal images, we performed a deconvolution analysis before making their surface 3D rendering (Figure 7B,C), to better identify the individual fluorescent cells in the thickness of the invaded area (Figure 7C).

#### 2.4.2. Single Cell-Tracking

**Figure 8:**
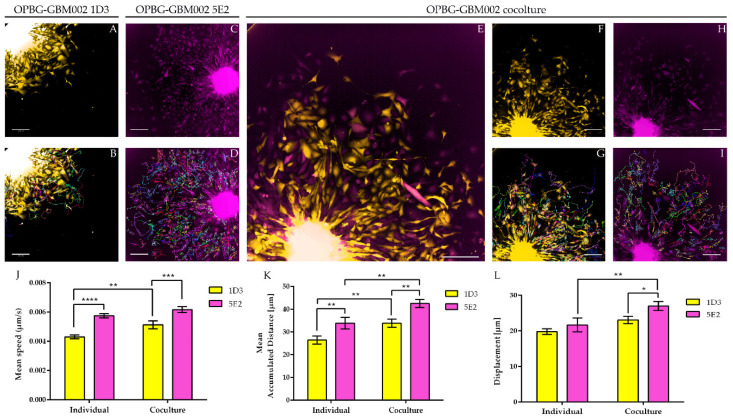
Single cell-tracking—3D migration assay. Representative fluorescent images of OPBG-GBM002 single cell-derived clones 1D3 ((**A**), m-Orange) and 5E2 ((**C**), Venus) 3D migration assays are shown as mono-culture and as co-culture 1:1 ((**E**), overlay of m-orange and Venus; (**F**), m-Orange and (**H**), Venus). Images were acquired on the Operetta CLS (PerkinElmer) every 30 min for 96 time points and shown are the images relative to time point 40. Scale Bar = 200 μm. Single cell-tracking was performed using the Harmony software (PerkinElmer) and is represented with the lines and arrows overlayed on the fluorescent images ((**B**) and (**D**) for 1D3 and 5E2 in mono-culture, respectively; (**G**) and (**I**) for 1D3 and 5E2 in co-culture, respectively). Mean speed (**J**), mean accumulated distance (**K**) and displacement (**L**) were analyzed with Harmony software. Data are mean ± SD, *n* = 3. (****) *p* < 0.0001; (***) *p* < 0.001; (**) *p* < 0.01; (*) *p* < 0.05.

As mentioned above, a useful application of the Multifluorescent Marking Technology is the study of direct cell–cell interactions. Although the Operetta CLS presents some limitations in the separation of the 6 fluorescent proteins and in the definition of the optical barcodes of the single cell-derived clones, it can certainly be used for analyzing several aspects of their phenotypic characterization also in live imaging, as exemplified in Figure 8 for the single-cell tracking of 3D migration assays. Two individual single cell-derived clones, 1D3 and 5E2, both derived from the multifluorescent bulk cell line OPBG-GBM002, were utilized in 3D migration assays individually (Figure 8A,C, and Videos S1 and S2, respectively) and in co-culture (Figure 8E,F,H and Video S3). Based on the optical barcodes previously determined using the TCS SP8X confocal microscope, clones were finely distinguished and imaged on the Operetta CLS based on the expression of Venus for 5E2 and m-orange for 1D3. Using the Harmony software on the Operetta CLS (Perkinelmer), the single cell-tracking was performed (Figure 8B,D,G,I) for the clones in mono and co-culture and mean speed, mean accumulated distance, and displacement (Figure 8J–L, respectively) were analyzed. The clone 5E2 (Venus) appeared generally more “*motile*” than the clone 1D3 (m-Orange). Interestingly both clones seemed to benefit from the co-culture condition as they showed significantly higher speed (1D3), accumulated distance ((1D3 and 5E2) and displacement (5E2) when compared to their mono-cultures (Figure 8J–L).

#### 2.4.3. Ex Vivo 3D Invasion on Organotypic Brain Slice

In addition to the in vitro 3D invasion model, we used also the ex vivo whole brain organotypic brain slice (OBS) culture model [23,24].

In order to co-culture OBS with the DIPG cells, the slices were cultured with the same stem cell medium used to grow the DIPG cells. We first verified that in this culture condition, the mouse brain cytoarchitecture was preserved. In order to do so, we looked for the presence of different cell types of the cerebral tissue including neurons, microglia, oligodendrocytes and astrocytes and confirmed the expression of their associated markers (Figure S8) at day 0 (immediately after slice preparation) and at 14 days (end-point of the co-culture with DIPG cells), indicating that the OBS were not affected by the stem cell medium.

**Figure 9:**
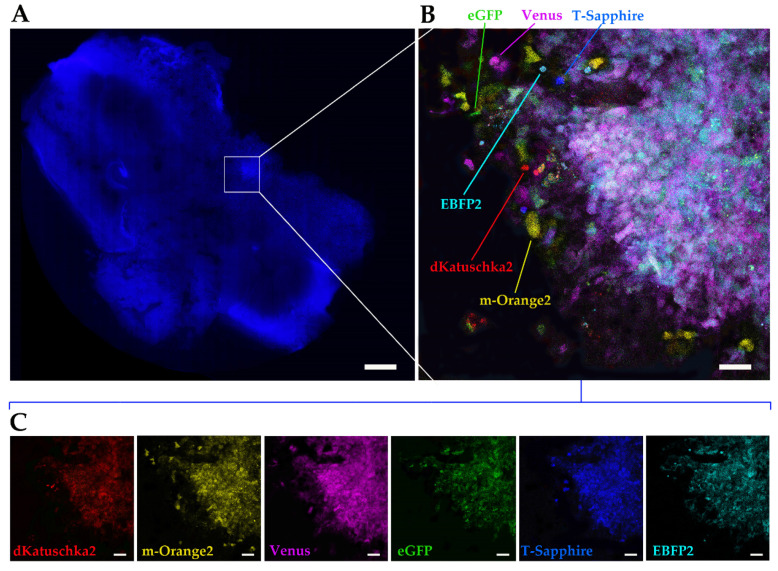
Multifluorescent ex vivo 3D tumor invasion on OBSc. (**A**) Hoechst staining of a representative whole brain organotypic slice cultures (OBSc), encompassing pons and medulla, was acquired at a digital slide scanner (Nanozoomer S60, Hamamatsu). (**B**,**C**) Representative images of multifluorescent bulk OPBG-DIPG002 cell invasion on cleared OBS are shown. Images were acquired on a Leica AOBS-SP8X confocal microscope, after tissue clearing to reduce brain tissue autofluorescence. Overlay confocal image (**B**) showing single fluorescent scattered cells propagating outside the central area. The fluorescence of invading cells is shown also on the split panel (**C**) in red (dKatushka2), yellow (m-Orange2), magenta (Venus), green (eGFP), blue (T-Sapphire) and cyan (EBFP2) channels. Scale bars: 1mm in (**A**), 50µm in (**B**,**C**).

From the multifluorescent OPBG-DIPG002 bulk cell line, we generated neurospheres of 400–450 μm of diameter, which were implanted in the pontine area, one neurosphere by brain slice. Seven days after implantation, the OBS were fixed and Hoechst staining was performed to visualize the cell nuclei. Mosaic images of the co-cultured DIPG/OBS were acquired on a digital slide scanner (Nanozoomer S60, Hamamatsu, Shizuoka, Japan) to easily assess the OBS integrity and identify the DIPG cell invasion areas (Figure 9A).

Prior to acquire images with the confocal microscope and get a better and more detailed view of the multifluorescence DIPG cell invasion area, we performed tissue clearing [25]. This was done to reduce the brain tissue autofluorescence that was observed in preliminary experiment (data not shown). Following that, the confocal images were acquired (Figure 9B,C) and displayed the multifluorescent DIPG cells that invaded the brain tissue. We could discriminate single fluorescent scattered cells propagating outside the central area of greatest cell density (Figure 9B). It was possible to recognize all of the six fluorescences on both, the overlay image and the split panel. Our data suggest that the Multifluorescent Marking Technology combined with 3D ex vivo OBS culture is a feasible model to study more in-depth the DIPG intratumor heterogeneity, offering specific insights into the invasion process and the potential involvement of the microenvironment (Figure 9C).

## 3. Discussion

The genetic and phenotypic intratumor heterogeneity may represent one of the most challenging obstacles in the development of effective therapies for cancer. Intratumor heterogeneity has been shown to be strictly associated to therapeutic resistance and, as such, may be a major cause of tumor progression and disease relapse [26,27,28,29].

pGBM and DIPG are known to be constituted by heterogeneous cell subpopulations coexisting within the same tumor and cooperating into a functional network, causing resistance to drug treatments, and increasing the aggressive phenotype [12]. Moreover, a functional network is also present between the tumor and the normal brain microenvironment, in particular with its neuronal compartment, which actively contributes to promote glioma progression [14,15].

Understanding the mechanisms of cell–cell communication taking place within the tumor and with its microenvironment could lead to the identification of new, more effective therapeutic strategies to treat these devastating diseases.

We sought to use the Multifluorescent Marking Technology to establish the tools that will enable future investigations into the cellular and molecular mechanisms of intra-tumor heterogeneity in pGBM and DIPG.

Originally the RGB Marking technology was used to study the heterogeneity and the clonal dynamics in vitro and in vivo, in osteosarcoma [30], pancreatic adenocarcinoma [31], hepatocellular carcinoma [32], mammary adenocarcinoma [33], and neuroendocrine carcinoma [16].

Here, we focused on the application of the Multifluorescent Marking Technology and on the integration of different fluorescence analysis platforms, identifying their strengths and limitations, with the goal to understand how we could apply this technology to further expand our studies on pGBM and DIPG heterogeneity and clonal dynamics.

After the establishment of four pHGG patient primary derived cell lines, we generated the bulk multifluorescent populations using the approach initially reported by Weber et al., for the RGB marking technique [16,19], and with the six LeGO vectors used for the optical barcoding described by Momhe et al. [17].

To transduce our primary derived cell lines, we adapted the original protocol to accommodate the stem cell culture conditions, used for pGBM and DIPG cells. The neurosphere stem cell culture is generally accepted as the gold standard for primary glioma stem cells [34,35]. Recently, two studies have employed a reliable protocol for lentiviral cell transduction of primary DIPG cell lines cultured as neurospheres, by exposing the cells to FBS for a short period of time [36,37]. They have demonstrated that the short-term exposure of the cells to serum and a rapid return to serum-free conditions, improve the neurosphere transduction efficiency without inducing a change in their stem cell proprieties. However, in our case, we decided not to expose our primary derived cells to serum and to perform the lentiviral transduction on laminin-adherent stem cell culture condition [20]. This allowed us to obtain a good enough lentiviral cell transduction efficiency and at the same time, as the cells grew adherent, we could easily assess the different fluorescences during the initial stages of the multifluorescent bulk cell expansion, and during the processes of the generation and expansion of the single cell-colonies. It is important to note that these are rare tumors and often the primary cultures are established by small biopsy samples. Any type of culture will exert a selective pressure on the expanded cell lines. For this reason, we transduced our cells after passage 10 in order to work with cell lines more stable in their sub-clonal composition. We believe this has been maintained over time and over passages also after cell transduction as demonstrated by the phenotypic features (e.g., morphology, migration, invasion pattern) that the bulk cell lines and the single-cell-derived clones display.

To evaluate the transduction efficiency of the bulk cell lines, we used the flow cytometry. Our challenge has been to separate the six different fluorescences, which have distinct but, in some cases, overlapping excitation and emission wavelengths. Given the filter configuration of our flow cytometer (Table 2) and the close range of the wavelengths, we were able to discriminate only four out of the six fluorescent proteins. For each cell lines, we could determine the percentage of positive cells and the fluorescent intensity level for m-Orange2, dKatushka2, EBFP2, and T-Sapphire. However, we were unable to distinguish Venus from eGFP due to the strong overlap of their emission spectra, thus, preventing us from determining the transduction efficiency for these two fluorescent proteins. Our results are partially in contrast with what has been previously reported by Mohme et al., who have been able to separate the six different fluorescent proteins and perform the analysis of the clonal composition of the established glioblastoma cell line by flow cytometry [17]. The reason of the discrepancy between these results is the use, by Mohme et al., of a specific customized filter for Venus detection, which has been fundamental for their analysis and subsequent assignment of the optical barcodes for the cells and the derived clones.

Although we were not able to discriminate two out of the six fluorescent proteins, the flow cytometer has been essential in our study for the generation of the single cell-derived clones. Using a precise cell sorting strategy, we obtained single cell-derived clones positive for any of the six proteins as further confirmed with the acquisition of the cell images by confocal microscopy.

Moreover, differently from Mohme et al., where the flow cytometry was their main approach used to assign optical barcodes, our major interest was to visualize the multifluorescent bulk cells and the derived clones and based on that, set up the right models that would enable us to study the mechanisms of cell–cell interactions at different levels of complexity, over a long period of time.

To achieve this, we focused our image analysis using a freely tunable confocal microscope, the Leica TCS AOBS-SP8X laser scanning confocal microscope. This platform is equipped with a tunable white light-laser (WLL) and a tunable beam splitter, which enabled us to precisely distinguish each individual fluorescent protein, for all the four multifluorescent bulk cell lines. This has been possible based on the pulsed nature of the WLL that allows to specify any excitation wavelength between 470–670 nm, together with a fast beam splitting device. The AOBS can select simultaneously up to eight discrete laser lines, even with narrow distances between excitation lines, with a 1nm precision. Furthermore, we used the WLL-AOBS system in conjunction with hybrid detectors, thus obtaining gated removal of autofluorescence and reflected light. All these devices allowed us to reach maximum detection sensitivity results.

In addition, to clearly characterize the multifluorescent pGBM and DIPG primary cell lines, the WLL-AOBS confocal microscope has been fundamental for determining the OB of the single cell-derived clones.

Of note, the OBs could be further refined taking in consideration not just the combination of fluorescences, but also the fluorescent intensity of each of the protein expressed. As clearly shown by Gomez-Nicola D. et al., cells are randomly transduced by one or more lentiviral vectors. Also, the viruses may integrate at different sites leading to different expression levels. In addition, multiple hits from the same vector may lead to additional expression variation [38]. The combination of these factors determines the unique hues associated with each clone. An important point to note is that the metabolic state and the cell cycle phase can also affect the fluorescent protein expression level leading to variations in fluorescence intensity in cells from the same clone [39].

Next, we used the Operetta CLS developed by PerkinElmer. The Operetta CLS is an automated microscope for high-content/high-throughput image acquisition and analysis. It can acquire and analyze fluorescence, brightfield and digital phase images as well as be used in direct or confocal mode. Also, it allows the acquisition of fixed and live images. It could represent an ideal instrument to study cell–cell interaction, communication, migration, single cell tracking and perform time laps experiments in a high-content manner. Although this platform may be equipped with different LED sources and as many combinations of excitation and emission band range selection filters, there are not very restrictive filters making the Operetta a not very versatile platform in the separation of multiple fluorescences as tested in our study. With the Operetta CLS, we could identify m-Orange2 and dKatushka2, but we were not able to discern Venus from eGFP and EBFP2 from T-Sapphire. This inability to discriminate some of the fluorescences, does not make the Operetta CLS suitable for the analysis of the multifluorescent bulk population of cells neither for the identification and assignment of unique OB for the single cell-derived clones. Since the Operetta CLS is a highly automated microscopy station, it does not have the same versatility and possibility of tuning the spectrum wavelengths as the SP8 AOBS platform, useful to perform both a fine separation of the emission spectra of the different fluorescent proteins and for the high imaging resolution. Therefore, a freely tunable or a spectral confocal microscope platform remains the ideal imaging platform to characterize the multifluorescent pGBM and DIPG bulk cell lines and to determine the OB of individual clones. On the other hand, a confocal microscope would not be ideal for a multi-well format (96-384 well plates), large scale experiments, where, for example, different combination of clone co-cultures are tested, or drug treatments are applied to different clones in co-cultures. Based on this, we believe that despite its limitations, an automated high-content imaging platform such as the Operetta CLS can still be very useful for studying their phenotypic characterization and acquiring a large amount of data. Moreover, although some fluorescences may not be well separated, using the exclusive expression of one or another fluorescent protein of the OBs, clones in co-culture could still be discriminated. All of this has been clearly exemplified in the single cell-tracking experiments performed with the two single cell-derived clones in mono- and co-culture. The live imaging and analysis performed on the Operetta CLS has readily provided insights into the phenotypic heterogeneity that characterize these cell populations in particular in terms of their motile capability and how this can be modulated by their direct cell–cell interactions.

Following the assessment in 2D culture, we exploited the application of the Multifluorescent Marking Technology on two different 3D invasion models, in vitro and ex vivo, and evaluated the suitability of our assays with the imaging capability of our systems.

We used a multifluorescent bulk DIPG cell line in vitro for the 3D invasion into Matrigel [21,22] and at the invasion assay end point, the images were acquired and analyzed on our Leica TCS AOBS-SP8X laser scanning confocal microscope. We were satisfied that despite the dense, packed 3D cell invasion, we could successfully distinguish each invading cell from the others, not only at the periphery but also in the more central area of the tumor invasion, discriminating the individual fluorescences. In particular, acquiring in Z-stack and performing a deconvolution analysis for the 3D rendering, allowed us a very clear detection of the individual fluorescent cells in the thick Matrigel sample.

Finally, we utilized the ex vivo OBS model [23] to provide a more physiological brain-like microenvironmental context for our DIPG cells to invade in. The same multifluorescent DIPG cell line used for the 3D invasion into matrigel, was implanted on OBSs. The OBSs themselves can determine some autofluorescence, which, together with the multicolor cells and the thickness of the slices, can make more challenging the separation of the different fluorescences, even using our confocal microscope. To achieve that and obtain a clearer view of the implanted, infiltrated multifluorescent DIPG cells, we performed tissue clearing, which enabled to distinguish all the six fluorescences.

Overall, our study demonstrates the applicability of the Multifluorescent Marking Technology to patient primary cultures of pGBM and DIPG, from 2D to more complex 3D environments. We have explored and integrated multiple fluorescent analysis platforms, highlighting their strengths and limitations in the analysis of such technology. In conclusion, with the specific platforms we had at our disposal, we have successfully used our Leica TCS AOBS-SP8X laser-scanning confocal microscope to perform high resolution imaging of the multifluorescent bulk cell lines, in 2D and 3D, as well as precisely define the OB of the single cell-derived clones. The BD FacsAria^TM^ III flow cytometer, despite its limitations in the separation of 2 out of 6 fluorescent proteins, has been critical with the adopted cell sorting strategy, for the generation of the single cell-derived clones. Finally, the Operetta CLS has also shown limitations in the separation of the 6 fluorescent proteins, but its fluorescence, automated, confocal, high-content imaging capability, has demonstrated its applicability for an experiment of phenotypic characterization of the single-cell-derived clones and their direct cell–cell interactions.

Our study has contributed on establishing the tools to study further the intratumor heterogeneity and the interclonal interactions in pGBM and DIPG.

## 4. Materials and Methods

### 4.1. Cell Cultures

pGBM and DIPG primary patient-derived cell lines were established either immediately after collection (biopsy or resection) or from live cryopreserved tissue. Cell lines were established from fresh tissue, first minced with a sterile scalpel followed by enzymatic dissociation with LiberaseTL (Roche Life Science, Penzberg, Germany) for 20 min at 37 °C in the presence of 1 U/mL DNase I (Thermofisher Scientific, Waltham, MA, USA) (shaking every 5 min). After enzyme neutralization and centrifugation, minced tissue was resuspended first in 1 mL HBSS 1× for further mechanical dissociation, then in 5 mL. After that, the minced tissue was filtered through a 70 μm filter. Cell cultures were established under stem cell conditions as two-dimensional (2D) adherent cultures on laminin (10 μg/mL) (Millipore, Burlington, MA, USA) and/or three-dimensional (3D) as neurospheres (NS). Hemispheric pGBM cell lines OPBG-GBM002 (histone WT), OPBG-GBM001 (H3.3 G34R), and DIPGs OPBG-DIPG002 (H3.3 K27M) and OPBG-DIPG004 (H3.1 K27M) were cultured in a serum-free medium designated as “Tumor Stem Media (TSM)”, consisting of 1:1 Neurobasal(-A) (Invitrogen, Carslsbad, CA, USA), and DMEM: F12 (Invitrogen), supplemented with Anti-mycotic/Anti-biotic, HEPES, NEAA, GlutamaX, Sodium Pyruvate (Invitrogen) and B27(-A) (Invitrogen, Carlsbad, CA, USA), human bFGF (20 ng/mL), human EGF (20 ng/mL), human PDGF-AA (10 ng/mL) and PDGF-BB (10 ng/mL) (Peprotech, Rocky Hill, NJ, USA) and heparin (2 ng/mL) (Stem Cell Technologies, Vancouver, BC, Canada). The cell authenticity was verified using short tandem repeat (STR) DNA fingerprinting by Eurofins Genomics (Table 5) and certified mycoplasma-free.

HEK293T cells (ATCC, Manassas, VA, USA) were used for lentiviral particle production and cultured in DMEM high glucose (Euroclone, Pero, Italy) including 10% Fetal Bovine Serum (FBS) (Invitrogen, Carlsbad, CA, USA) and 1% pen-strep (Euroclone, Pero, Italy).

All patient samples were collected under full Research Ethics Committee approval.

### 4.2. DNA Extraction and Sanger Sequencing

DNA was extracted from multifluorescent primary cell pellets following the DNeasy Blood & Tissue kit protocol (Qiagen, Hilden, Germany) and was measured on the Nanodrop 2000 (Thermofisher, Waltham, MA, USA). PCR for *H3F3A* and *HISTI1H3B* was carried out using primers obtained from Sigma Aldrich (St. Louis, MO, USA). Products were purified using NucleoSpin Gel and PCR Clean-up (Macherey-Nagel, Dueren, Germany), quantified on a Nanodrop 2000 (Thermo Scientific, Waltham, MA, USA) and subjected to bidirectional sequencing using BigDye Terminator v3.1 (Applied Biosystem™, Foster City, CA, USA). After purification with Nucleoseq (Macherey Nagel, Dueren, Germany), capillary sequencing was done on a 3500 Genetic analyzer (Applied Biosytem™). Sequences were analyzed using the Mutation Surveyor (SoftGenetics, State College, PA, USA) and manually with FinchTV (Geospiza, Seattle, WA, USA).

### 4.3. Lentivirus Particles Production

The third-generation HIV-1-derived self-inactivating lentiviral gene ontology vectors (LeGOs) were used for stable transduction of pGBM and DIPG cells. The vectors were previously described by Mohme et al. [17]: LeGO-G2 (expressing eGFP, plasmid 25917; Addgene, Watertown, MA, USA), LeGO-V2 (expressing Venus, plasmid 27340; Addgene, Watertown, MA, USA), LeGO-EBFP2 (expressing EBFP2, plasmid 85213; Addgene), LeGO-S2 (expressing T-Sapphire, plasmid 85211; Addgene), LeGO-mOrange2 (expressing mOrange2, plasmid 85212; Addgene) and LeGO-dKatushka2 (expressing dKatushka2, plasmid 85214; AddgeneLentivirus particle production was performed as previously described [19], with some modifications. Briefly, HEK293T cells were seeded, and transient transfection, with Lipofectamine 2000 (Invitrogen) in according to the manufacturer’s instructions, was performed using separately each one of the LeGO plasmids and the third-generation packaging plasmids pMDLg/pRRE (plasmid 12251; Addgene), pRSV-Rev (plasmid 12253; Addgene) and pMD2.G (plasmid 12259; Addgene). Cells were incubated for 12 h and then the medium was changed. After 48 h, the supernatant with the lentivirus particles was collected.

### 4.4. Lentivirus Concentration

Lentiviral supernatants were filtered with 0.22 µm Steriflip-GV (Millipore). After that, the lentiviral particles were concentrated using the Lenti-X concentrator (TakaraBio, Shiga, Japan) according to the manufacturer’s protocol. 1 volume of Lenti-X concentrator was combined with 3 volumes of supernatant and then mixed by gentle inversion. The mixture was incubated overnight at 4 °C. The day after, the mixture was centrifuged at 1500× *g* for 45 min at 4 °C. After centrifugation, the supernatant was carefully removed, and the lentiviral particle pellet was resuspended in 1/10 of the original volume in Phosphate-Buffered Saline (PBS) (Euroclone, Pero, Italy).

### 4.5. Titration and Titer Calculation of Lentivirus Vector

The viral particle titration was performed as previously described [19]. Briefly, 5 × 10^4^ HEK293T cells were seeded in 24-well plate and following cell attachment, the polybrene (8 μg/mL) (Sigma-Aldrich, St. Louis, MO, USA) was added to the medium to increase the transduction efficiency. For each lentivirus, different lentiviral particle amounts (1; 10 and 100 μL) were added in three different wells, the plate was centrifuged for 1 h at 1000× *g* at 25 °C and then placed in an incubator at 37 °C, 5% CO_2_ for 72 h.

For the titer calculation, the transduced HEK293T cells were analyzed at the flow cytometer and then the titer calculation was done following the previously described method [19].

### 4.6. Transduction of Primary Patient-Derived pGBM and DIPG Cell Lines

The pGBM and DIPGs primary cell transduction was performed as previously described [19], with some modifications. All cell lines were transduced between passage 10–14. 5 × 10^4^ target cells were seeded in a laminin pre-coated 24-well plate and incubated at 37 °C and 5% CO_2_. The day after, the media was changed, and the polybrene (8 μg/mL) was added. The lentivirus particles from the six vectors were added to the cells altogether as well as individually. As already reported for primary cells [19], our primary cultures were difficult to transduce. For this, the amount of lentivirus used was 100× more concentrated than the titer calculated for our cells. After adding the lentivirus particles, the plate was centrifuged for 1 h at 1000× *g* at 25 °C, incubated at a 37 °C, 5% CO_2_ and 48 h later, the medium was changed. During the following week, the fluorescence associated with a successful cell transduction was checked at a Leica DMi8 (Leica Microsystems, Wetzlar, Germany) fluorescence microscope. The bulk cell lines were then expanded.

### 4.7. FACS Analysis and Sorting of Multifluorescent Bulk Population Primary Cell Lines

To obtain a bulk cell line composed of only transduced cells, the non-marked cells were sorted out using BD FacsAria^TM^ III (BD Bioscience, San Jose, CA, USA) flow cytometer with cell-sorting capability. To analyze the transduction efficiency of multifluorescent bulk DIPG and pGBM cells, we used the BD FacsAria^TM^ III (BD Bioscience, USA) flow cytometer with a specific filter configuration (Table 2). The percentage of fluorescently positive cell was determined with FACSDiva software 8.0 (BD Bioscience).

### 4.8. Confocal Microscopy

Confocal microscopy was performed on a Leica TCS AOBS-SP8X laser-scanning confocal microscope (Leica Microsystems) equipped with tunable white light laser (WLL, 470–670 nm of wavelength) source, 405 nm diode laser, 3 photomultiplier tubes, 2 HyD detectors and an acousto-optical beam splitter (AOBS) that allowed the separation of multiple fluorescences. Sequential confocal images were acquired using HC PL APO CS 10X/0.40 or HC PL APO CS2 20X/0.75 objectives (Leica Microsystems) with a 1024 × 1024 format, scan speed 400–600 Hz, and z-step size of 1 µm. Fluorochromes unmixing was performed by the acquisition of an automated-sequential collection of multi-channel images to reduce spectral crosstalk between channels.

Maximum intensity projection (MIP) of z-series and complete mosaic image using Tile Scan function were performed by LASX (Leica Microsystems) software; deconvolution analysis (HyVolution2 software, Leica Microsystems) was applied to z-stacks to improve contrast and resolution of confocal raw images, then deconvoluted images were imported into LASX 3D (Leica Microsystems) software to obtain their surface 3D rendering. Tables of images were processed using Adobe Photoshop CS4 software (Adobe Systems Inc., San Jose, CA, USA).

### 4.9. Operetta CLS Image Acquisition

The expression of the six different fluorescent proteins in the bulk cell lines and in the single cell-derived clones was performed on an Operetta CLS (PerkinElmer, Waltham, MA, USA), equipped with eight emission LED filters with the emission/excitation configuration described in Table 4. Image acquisitions were performed using the 20× objective (numerical aperture 0.4).

### 4.10. Establishment of Single Cell-Derived Clones

Bulk multifluorescent cell lines were single cell-flow sorted using a BD FacsAria^TM^ III instrument (BD Bioscience) where single cells were sorted unbiased by any marker expression directly into the inner 60 wells of 5 laminin (Millipore) pre-coated flat-bottom 96-well plates (PerkinElmer). Single cells were dropped in 100 μL/well of the same medium as described above. The outer 16 wells were filled in with 200 μL/well of PBS to avoid evaporation of medium. After single cell-flow sorting, the plates were incubated at 37 °C, 5% CO_2_, and colonies monitored as previously described [12]. Once weekly cells were refed with 25 μL of medium/well and plates scanned on a CeligoS cytometer (Nexcelom Bioscience, Lawrence, MA, USA) using the Confluence application, to evaluate colony growth. Single cell-derived colonies were detached using Accutase (Euroclone, Pero, Italy) when they achieved approximately 60–80% confluency and collected for further expansion to stably derive single cell-derived clones.

### 4.11. Invasion Assay

3D invasion assays were performed as previously described [12,21,22], with some modifications. The bulk cells were detached and counted. For single NS, 1000 cells/well in 100 μL were dispensed into ultra-low attachment (ULA) 96-well round-bottom plates (Corning, New York, NY, USA) using a multichannel pipette. The plates were transferred to an incubator (37 °C, 5% CO_2_) and three to four days later, when the neurospheres reached a size of 300–350 μm in diameter, the invasion assay was performed. A total of 50 μL medium was removed from each well and then, using ice-cold tips, 50 μL of Matrigel was gently dispensed into the ULA plates, which were then incubated at 37 °C, 5% CO_2_. 96 h later, the invasions were fixed with 4% paraformaldehyde (PFA) overnight at 4 °C. The day after, the fixed neurospheres were washed 3 times with PBS for 30 min and then images acquired at the confocal microscope. Images were processed using Adobe Photoshop CS4 software (Adobe Systems Inc.).

3D migration assays were performed as previously described [12,22] with some modifications. The 5E2 and 1D3 single cell-derived clone cells from the OPBG-GBM002 multifluorescent bulk cell line, were seeded into 96-well round-bottom ULA plates (Corning) at 1000 cells/well either in monoculture or in co-culture (50:50), and allowed to form a single NS per well. When the NS reached a size of 250–3000 μm in diameter, the migration assay was performed. Briefly, flat-bottomed 96-well plates (PerkinElmer) were coated for 2 h at RT with 50μL/well of 125μg/mL Matrigel (Corning) in culture medium in the absence of growth factors. Once coating was completed, a total of 200 μL/well of culture medium was added to each well. A total of 50μL medium was removed from ULA 96-well round-bottom plates containing NS, the remaining medium including the NS were transferred onto the matrigel pre-coated plates and cell let migrate for 48h. For live imaging experiments of the single-cell tracking, automated fluorescent image acquisition was performed at the Operetta CLS (PerkinElmer, Waltham, MA, USA) every 30 min for 96 time points, starting from 24 h after the migration assay was set up. In order to clearly distinguish the two clones, based on their optical barcodes, the fluorescent signal for m-orange was acquired for 1D3 and Venus for 5E2. The Harmony software (PerkinElmer) on the Operetta was used to calculate the mean speed, mean accumulated distance, and mean displacement from *n* = 3. Two independent experiments were performed.

### 4.12. Whole Brain Organotypic Slice Preparation and Co-Culture with Multifluorescent DIPG NS

Whole brain organotypic slices (OBSc)—encompassing pons and medulla—were prepared from CD1 mice pups (postnatal day 6–7) (Charles River, Wilmington, MA, USA) as previously described [23,24], with some modifications. In brief, mice were decapitated, and brains rapidly dissected and placed in ice-cold artificial cerebrospinal fluid (ACSF) containing (in mM): 126 NaCl, 3.5 KCl, 1.2 NaH_2_PO_4_, 1.2 MgCl_2_, 2 CaCl_2_, 25 NaHCO_3_ and 11 glucose (pH 7.3), saturated with 95% O_2_ and 5% CO_2_. The brain was then embedded in 3% SeaPlaque™ agarose (Lonza, Basel, Switzerland) in PBS and 300 µm thick sagittal slices were cut on a vibrating microtome (Campden Instruments, Sileby, UK), constantly cooled and oxygenated. We obtained around six slices, complete of pons and medulla, from each brain. Each slice was transferred onto a porous membrane (0.45 µm pore size, Millipore) placed on a Millipore culture insert (Millipore), inserted into six-well plates with 1.2 mL of pGBM/DIPG culture medium/well, where the inserts were placed. The slices were incubated at 35 °C, 5% CO_2_ for 7 days before the experiments to allow the inflammatory reaction following the mechanical procedure to subside. Following the first day of culture, the medium was replaced with fresh medium and, from that time, changed twice a week.

Some slices were processed immediately at the day of preparation (day 0) or two weeks after the preparation (day 14) for immunofluorescence for the cytoarchitecture characterization: brain slices were fixed with 4% PFA for 1 h at room temperature (RT) and rinsed twice with PBS. Slices were then permeabilized with 1% Triton in PBS for 90 min, blocked with 10% normal goat serum (NGS) + 1% bovine serum albumin (BSA) + 0.1% Triton in PBS for 1 h at RT, incubated with AffiniPure F(ab’)2 fragment goat anti-mouse IgG (H+L) 10 µg/mL (Jackson ImmunoResearch, West Grove, PA, USA) in PBS for 2 h and a half at RT and incubated with the primary antibodies over night at 4 °C. The antibodies were diluted in 2% NGS + 1% BSA in PBS at the following concentrations: rabbit anti-Glial Fibrillary Acidic Protein 1:500 (Z0334, Dako, Jena, Germany), mouse anti-CNPase 1:400 (clone 11-5B, Millipore, USA), rabbit anti-Iba1 1:1000 (FUJIFILM Wako Pure Chemical Corporation, Japan), mouse anti-NeuN 1:500 (clone A60, MAB#377, Millipore).

The day after, the slices were rinsed three times with 0.1% Triton in PBS and incubated over night at 4 °C with the secondary antibodies diluted in 2% NGS + 1% BSA in PBS at the following concentrations: goat anti-rabbit IgG (H+L) Alexa Fluor^®^ 488—conjugated and goat anti-mouse IgG (H+L) Alexa Fluor^®^ 555—conjugated, 1:500 (ThermoFisher Scientific). Slices were rinsed three times with 0.1% Triton in PBS, and nuclei were counterstained with Hoechst33342 (Invitrogen) in PBS for 45 min at RT, rinsed again with PBS and mounted on a slide. Images of the whole slices (4×) were taken on the Operetta CLS (PerkinElmer) while images of the hippocampal region at higher magnification (25×) were taken at Leica TCS-SP8X laser-scanning confocal microscope (Leica Microsystems).

7 days after slices were sectioned, OPBG-DIPG002 NS (1 neurosphere per slide) were implanted on the pontine area, and following 7 days of co-culture, during which DIPG cells had invaded, the brain slices were fixed with 4% PFA for 1 h at RT and rinsed twice with PBS. Slices were then permeabilized with 1% Triton in PBS for 90 min and incubated with Hoechst33342 (Invitrogen) 1:10,000 in PBS for 45 min at RT.

All animal procedures were under the European Communities Council Directive N. 2010/63/EU and the Italian Ministry of Health guidelines (DL 26/2014) and approved by the Italian Ministry of Health and by the local Institutional Animal Care and Use Committee (IACUC) at Istituto Superiore di Sanità (Rome, Italy; protocol n. D9997.N.BYG, 2019).

### 4.13. Tissue Clearing

To reduce brain tissue autofluorescence, whole-brain OSs at the end point of the co-culture with DIPG NS, were fixed over night at 4 °C in 4% PFA, washed three times in PBS, then embedded overnight at 4 °C in hydrogel monomer solution composed by 4% acrylamide (Sigma-Aldrich) supplemented with 0.25% of 2,2,-Azobis [2-(2-imidazolin-2-yl) propane] dihydrochloride initiator (VA-044, Fujifilm Wako Chemicals GmbH, Neuss, Germany) in PBS, as previously reported [25]. Sample-hydrogel polymerization was achieved by heating the samples at 37 °C for 3 h, then after PBS washes, samples were incubated with 4% SDS in 200mM boric acid in distilled water, pH 8.5 at 37 °C for 1 day, to passively remove membrane lipids. After clearing, samples were rinsed in PBS, mounted with PBS/glycerol 1:1, and imaged for high power-magnification of the multifluorescent invaded area, using a confocal microscope (Leica AOBS TCS-SP8X, Leica MicrosystemsAfter confocal imaging, samples were incubated in PBS to remove coverslips, then nuclei were counterstained with Hoechst33342 (Invitrogen) 1:10,000 in PBS for 45 min at RT. Finally, samples were mounted with PBS/glycerol 1:1 and acquired at the Nanozoomer S60 (Hamamatsu, Shizuoka, Japan) digital slide scanner platform.

### 4.14. Images Analysis of Fluorescence Intensity

The Mean Fluorescence Intensity and Fluorescence Intensity/single cell for each fluorescent protein were analyzed using ImageJ software (NIH, downloadable at http://rsbweb.nih.gov/ij/download.html). The analysis was carried out using 8-bit format digital images (TIFF format) acquired at the TCS AOBS-SP8X confocal microscope and at the Operetta CLS. For mean fluorescence intensity *n* = 4 randomly selected images per each cell line and clones were used. For Fluorescent intensity, *n* = 100 single cells were analyzed per each cell line and clones.

### 4.15. Statistical Analysis

All statistical analyses were performed using GraphPad Prism 6.0 (GraphPad software Inc., San Diego, CA, USA). The data are presented as mean ± SD (bar-plot) and as single value (dot-box-plot). *p*-value < 0.05 was considered to be statistically significant. Statistical analysis was performed using 2way ANOVA multiple comparison test.

## Figures and Tables

**Table 1:** Summary of demographic and clinical info of patient-derived cell lines.

Cell Line	Age (Years)	Sex	Procedure	Location	Diagnose	Mutation
OPBG-GBM002	11.4	M	Resection	Right fronto-temporal	GBM	Histone WT
OPBG-GBM001	12.3	M	Resection	Right fronto-temporal	GBM	*H3F3A* G34R
OPBG-DIPG002	5.7	F	Biopsy	Pons	DIPG	*H3F3A* K27M
OPBG-DIPG004	5.5	M	Biopsy	Pons	DIPG	*HIST1H3B* K27M

**Table 2:** FACS laser excitation and emission set up.

LeGO Vector	Excitation Laser Line (nm)	Dicroic Mirror LP (nm)	Emission Detection Range (nm)
EBFP2	405	-	BV421–450/40
T-Sapphire	405	502	BV510–510/50
eGFP	488	502	GFP–530/30
Venus	488	525	GFP–545/35
m-Orange2	561	570	PE–582/15
dKatushka2	561	735	PE-Cy7–780/60

**Table 3:** Configuration of Leica TCS AOBS-SP8 X confocal microscope.

LeGO Vector	Excitation Laser Line (nm)	Emission Detection Range (nm)
EBFP2	405	420–470
T-Sapphire	405	510–550
eGFP	470	480–510
Venus	515	540–570
m-Orange2	540	570–600
dKatushka2	594	>650

**Table 4:** Operetta CLS configuration.

LeGO Vector	Excitation Filter (nm)	Emission Filter (nm)
EBFP2	390–420	460–515
T-Sapphire	390–420	500–550
eGFP	435–460	500–550
Venus	460–490	500–550
m-Orange2	530–560	570–650
dKatushka2	530–560	655–705

**Table 5:** Cell lines authentication analysis.

STR Fingerprint										
Cell Culture	AMEL	CSF1PO	D13S317	D16S539	D21S11	D5S818	D7S820	TH01	TPOX	vWA
OPBG-GBM002	X, Y	10	11	14	30	12	10, 13	9, 9.3	8	18, 19
OPBG-GBM001	X,Y	12	9, 13	11, 12	30, 32.2	11, 12	8, 9	6	8	15, 17
OPBG-DIPG002	X, X	11, 12	12, 12	12, 12	29, 30	10, 12	9, 10	6, 6	8, 12	16, 16
OPBG-DIPG004	X, Y	10, 11	9, 12	11, 11	31.2, 32.2	11, 12	10, 10	8, 9.3	8, 11	14, 18

## Supplementary Materials

The following are available online at https://www.mdpi.com/1422-0067/21/18/6763/s1, Figure S1. Sanger sequencing chromatogram of pHGG patient primary derived-cell lines; Figure S2. Configuration and acquisition setup of the acousto-optical beam splitter (AOBS) SP8X confocal microscope used for the study; Figure S3. Additional multifluorescent bulk cell lines. Confocal microscope; Figure S4. Additional multifluorescent bulk cell line. Operetta CLS; Figure S5. Additional single cell-derived clones optical barcodes. Confocal microscope’ Figure S6. Additional single cell-derived clones optical barcodes. Operetta CLS; Figure S7. Images analysis of fluorescence intensity: confocal TCS SP8 and Operetta CLS; Figure S8. Cytoarchitecture characterization of organotypic brain slices; Video S1. 3D migration assay in mono-culture, clone 1D3; Video S2. 3D migration assay in mono-culture, clone 5E2; Video S3. 3D migration assay in co-culture 1:1.

## Abbreviations

pHGGs	Pediatric High-Grade Gliomas
pGBM	Pediatric Glioblastoma
DIPG	Diffuse Intrinsic Pontine Glioma
BMP	Bone Morphogenic Protein
LeGO	Lentiviral Gene Ontology
MOI	Multiplicity of Infection
OB	Optical barcode
MIP	Maximum Intensity Projection
OBSc	Organotypic brain slice culture
2D	Two-dimensional
3D	Three-dimensional
NS	Neurosphere
TSM	Tumor Stem Media
FBS	Fetal Bovine Serum
PBS	Phosphatase Buffered Saline
AOBS	Acousto-optical beam splitter
WLL	White Light-Laser
PFA	Paraformaldehyde
RT	Room Temperature
NGS	Normal Goat Serum
BSA	Bovine Serum Albumin
IACUC	Institutional Animal Care and Use Committee
STR	Short Tandem Repeat
