# Supplementary material for: Integration of Multiple Platforms for the Analysis of Multifluorescent Marking Technology Applied to Pediatric GBM and DIPG
Source: Int J Mol Sci. 2020 Sep 15;21(18):6763. doi: 10.3390/ijms21186763 (PMC7555235; doi:10.3390/ijms21186763)
Supplement: Supplementary file 1 [file ijms-21-06763-s001.zip › ijms-901129-si.pdf]

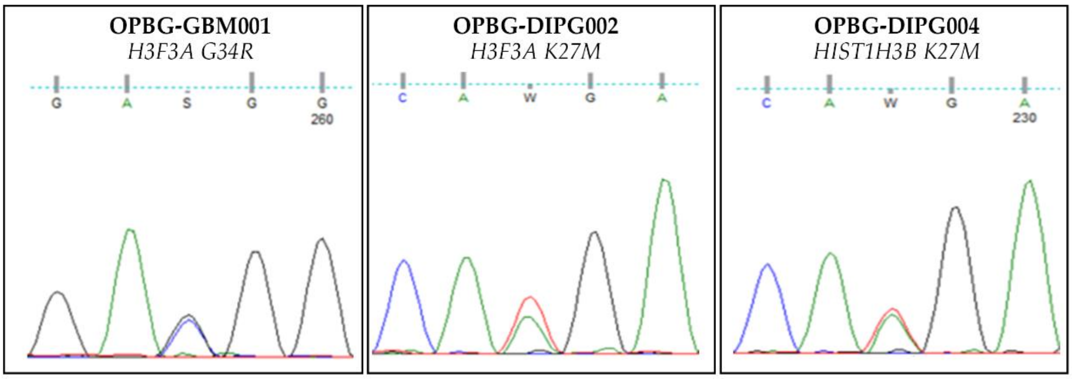

Figure S1. Sanger sequencing chromatogram of pHGG patient primary derived-cell lines. Sanger sequencing chromatograms show the mutations on histone genes: *H3F3A* G34R mutation in OPBG-GBM001, *H3F3A* K27M in OPBG-DIPG002 and *HIST1H3B* K27M in OPBG-DIPG004.

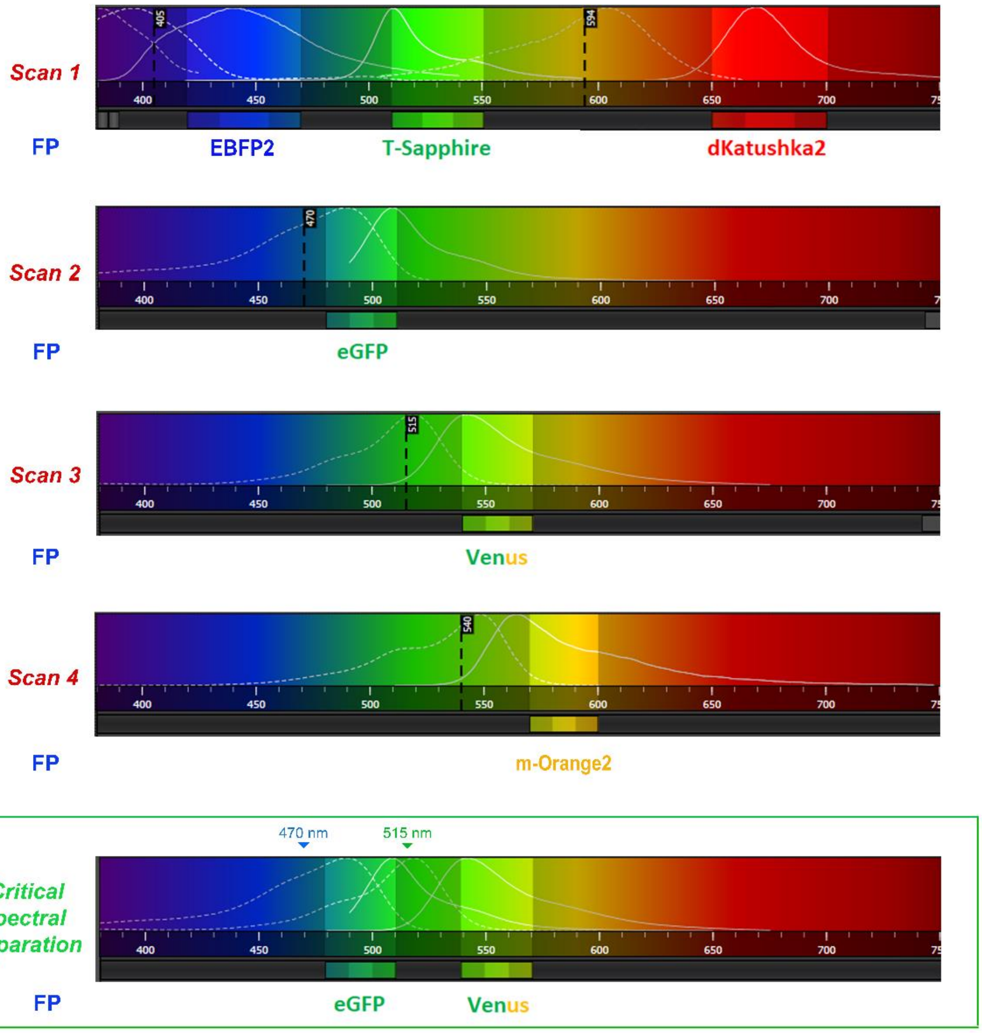

Figure S2. Configuration and acquisition setup of the acousto-optical beam splitter (AOBS) SP8X confocal microscope used for the study. Representative snapshots of acquisition scans for the six fluorescent proteins (FP) showing their excitation (dotted line) and emission (continuous line) spectra. In Scan 1, the simultaneous imaging of multiple fluorophores EBP2 and T-Sapphire, that are spectrally close, and dKatushka2 is possible by tuning both excitation and detection using 405 nm and 594 nm laser lines with almost null cross-excitation. Noteworthy, in Scans 2 and 3, the excitation

of eGFP and Venus that are spectrally close (as visualized by their adsorption and emission spectra in framed representative snapshoot), requires a sequential scanning with different excitation lines (488 nm and 514 nm, respectively) that allows an efficient spectral separation of the two close emission ranges (480–515 nm for eGFP, and 540–570 nm for Venus).

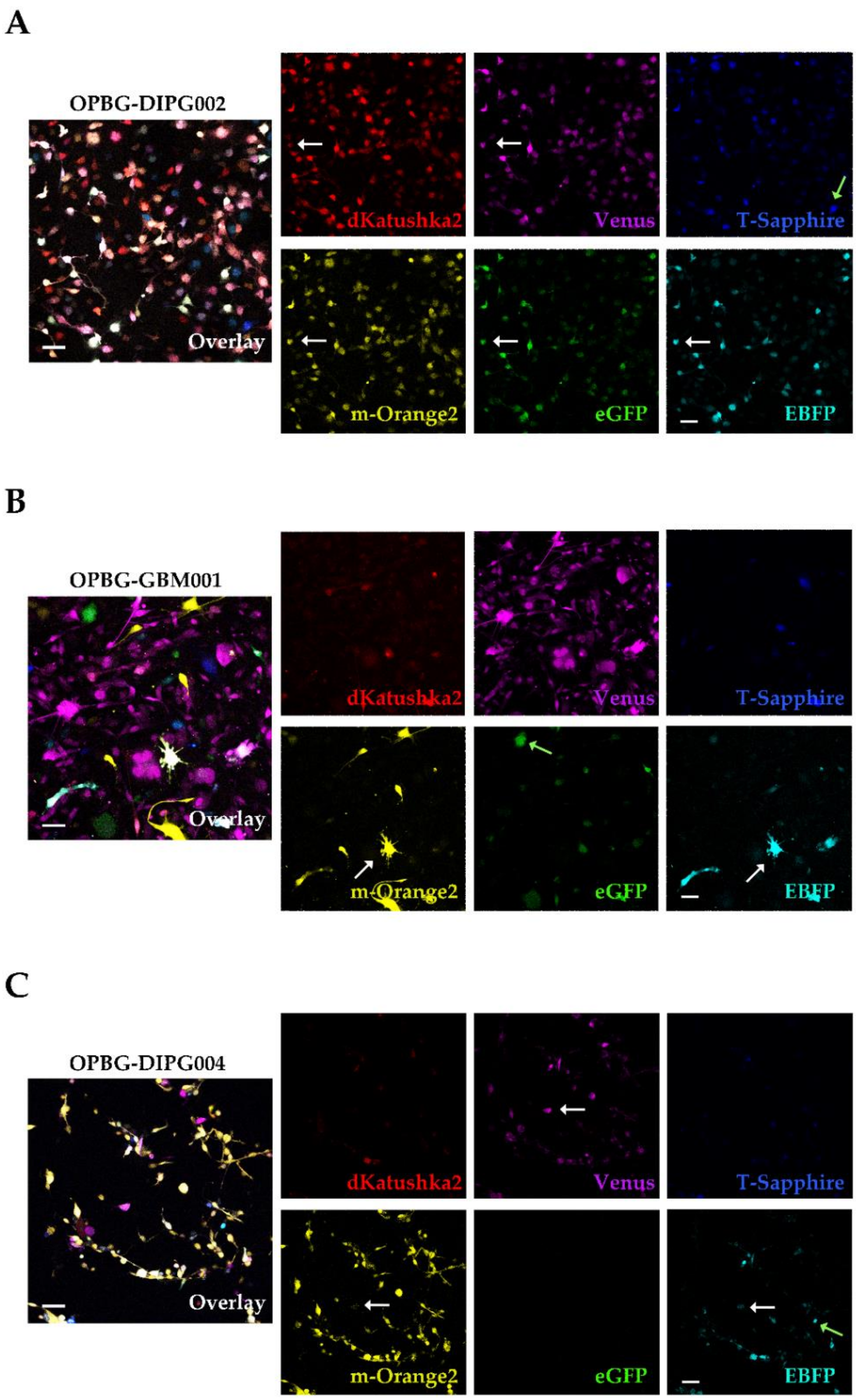

Figure S3. Additional multifluorescent bulk cell lines. Confocal microscope**.** OPBG-DIPG002 (**A**), OPBG-GBM001 (**B**) and OPBG-DIPG004 (**C**) primary cell lines were transduced using six different LeGO vectors. Fluorescence are shown in red (dKatushka2), yellow (m-Orange2), magenta (Venus), green (eGFP), blue (T-Sapphire) and cyan (EBFP2). In the images there are light green and white arrows to indicate respectively one-color and multicolor cells. All images were analyzed using confocal microscopy (Leica TCS ABOS-SP8X). Scale bars: 10μm

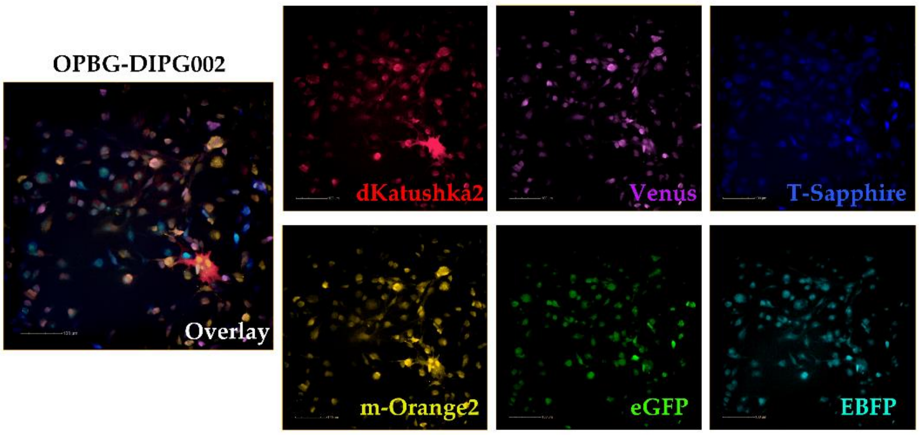

Figure S4. Additional multifluorescent bulk cell line. Operetta CLS**.** The OPBG-DIPG002 multifluorescent bulk population fluorescence are shown in red (dKatushka2), yellow (m-Orange2), magenta (Venus), green (eGFP), blue (T-Sapphire) and cyan (EBFP2). All images were analyzed using Operetta CLS fully equipped with 8 different emission filters. Scale bars: 100µm

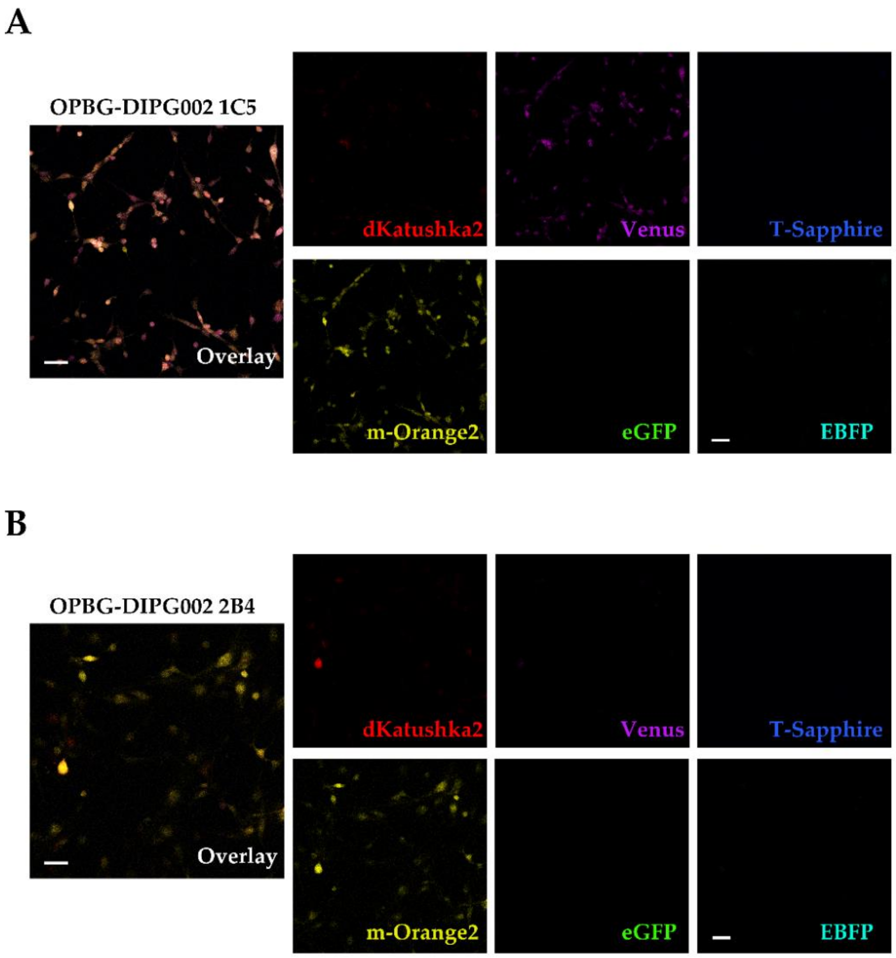

Figure S5. Additional single cell-derived clones optical barcodes. Confocal microscope. (**A**,**B**) Two different fluorescent OPBG-DIPG002 single cell-derived colonies are shown. The fluorescence resulting from OPBG-DIPG002 1C5 clone (**A**) are shown in red (dKatushka2), yellow (m-Orange2), magenta (Venus) and the fluorescence of OPBG-DIPG002 2B4 clone (**B**) are shown in red (dKatuhska2) and yellow (m-Orange2). Fluorescent image acquisition was performed using confocal microscopy (Leica TCS ABOS-SP8X). Scale bars: 10µm

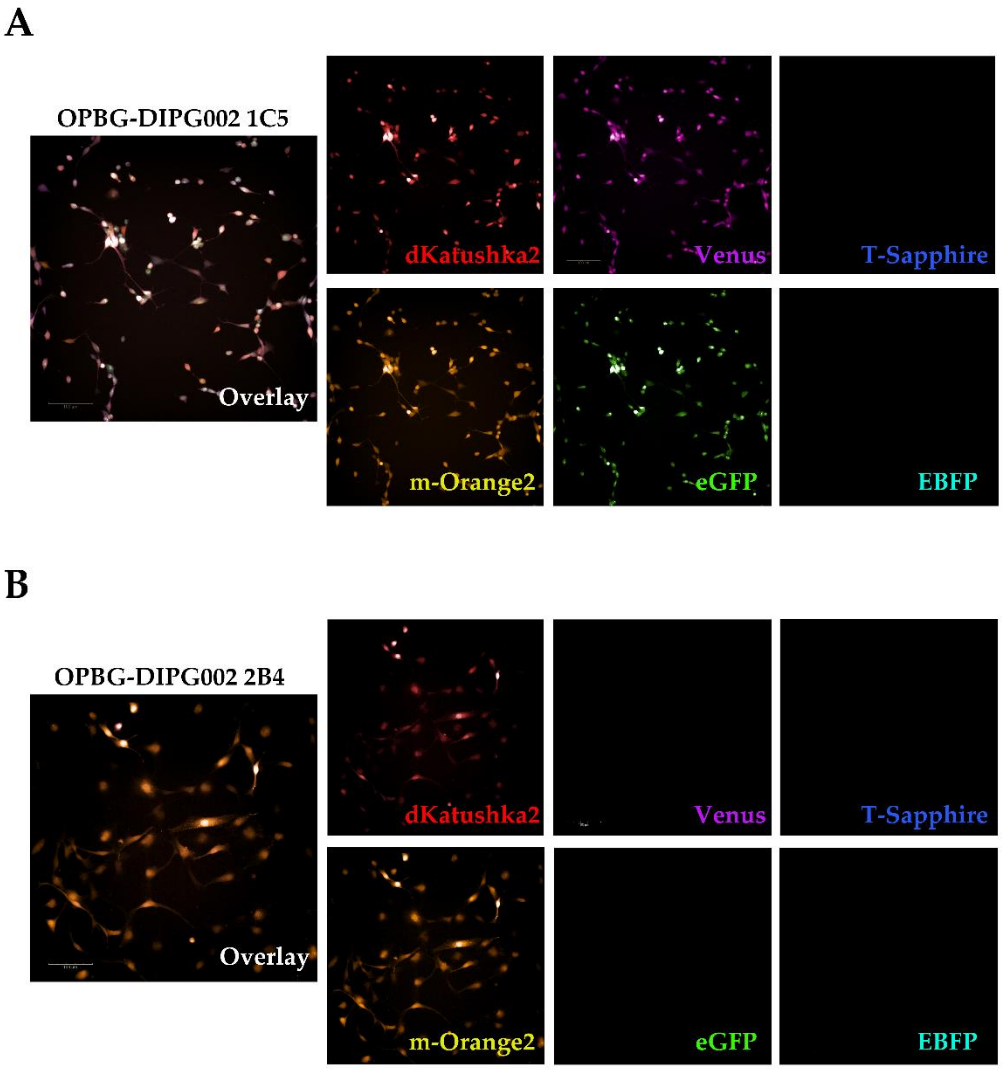

Figure S6. Additional single cell-derived clones optical barcodes. Operetta CLS. (**A**,**B**) Two different fluorescent OPBG-DIPG002 single cell-derived colonies are shown. The fluorescences resulting from OPBG-DIPG002 1C5 clone (**A**) are shown in red (dKatushka2), yellow (m-Orange2), magenta (Venus), green (eGFP) and the fluorescences of OPBG-DIPG002 2B4 clone (**B**) are shown in red (dKatushka2) and yellow (m-Orange2). All images were analyzed using Operetta CLS fully equipped with 8 different emission filters. Scale bars: 100µm

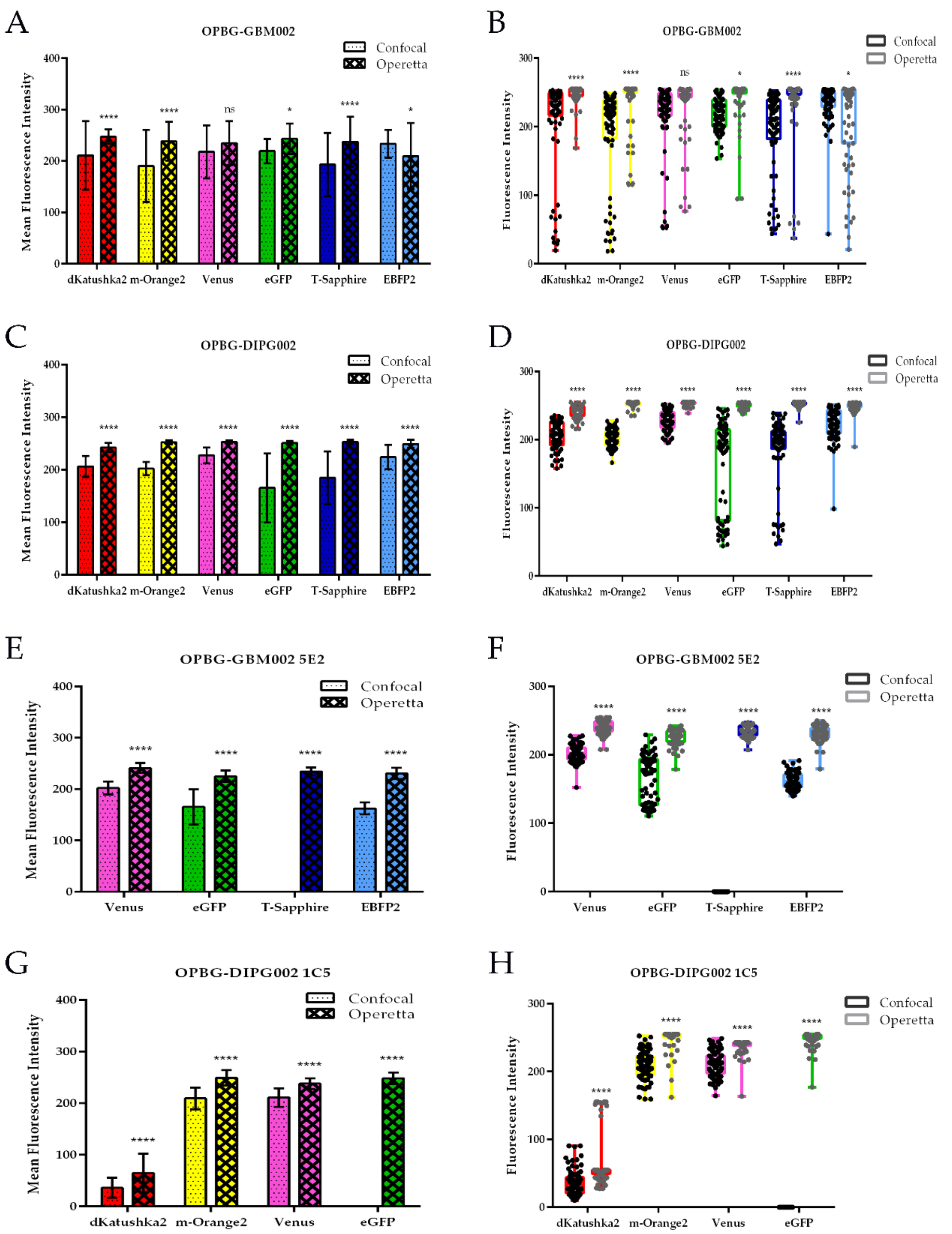

Figure 7. Images analysis of fluorescence intensity: confocal TCS SP8 and Operetta CLS. (**A–H**) The Mean Fluorescence Intensity and Fluorescence Intensity/single cell of each fluorescent protein, were analysed from OPBG-GBM002 (**A** and **B**) and OPBG-DIPG002 (**C** and **D**) multifluorescent primary cell lines and from OPBG-GBM 5E2 (**E** and **F**) and OPBG-DIPG002 1C5 (**G** and **H**) single cell-derived clones. Fluorescent images were acquired using TCS AOBS-SP8X confocal microscope and the Operetta CLS. The quantitative analysis was carried out using the ImageJ software side by side using digital images (TIFF format) from the two different platforms. Quantitative differences emerge between the two platforms due to the different resolution between optics being applied and the different width of the wavelength range that has been captured for each emission fluorescence. Results are mean ± SD of n=4 images analysed per cell line or clone for the mean fluorescence intensity and n=100 single cells analysed for the fluorescent intensity. (****) $p < 0.0001$; (*) $p < 0.05$.

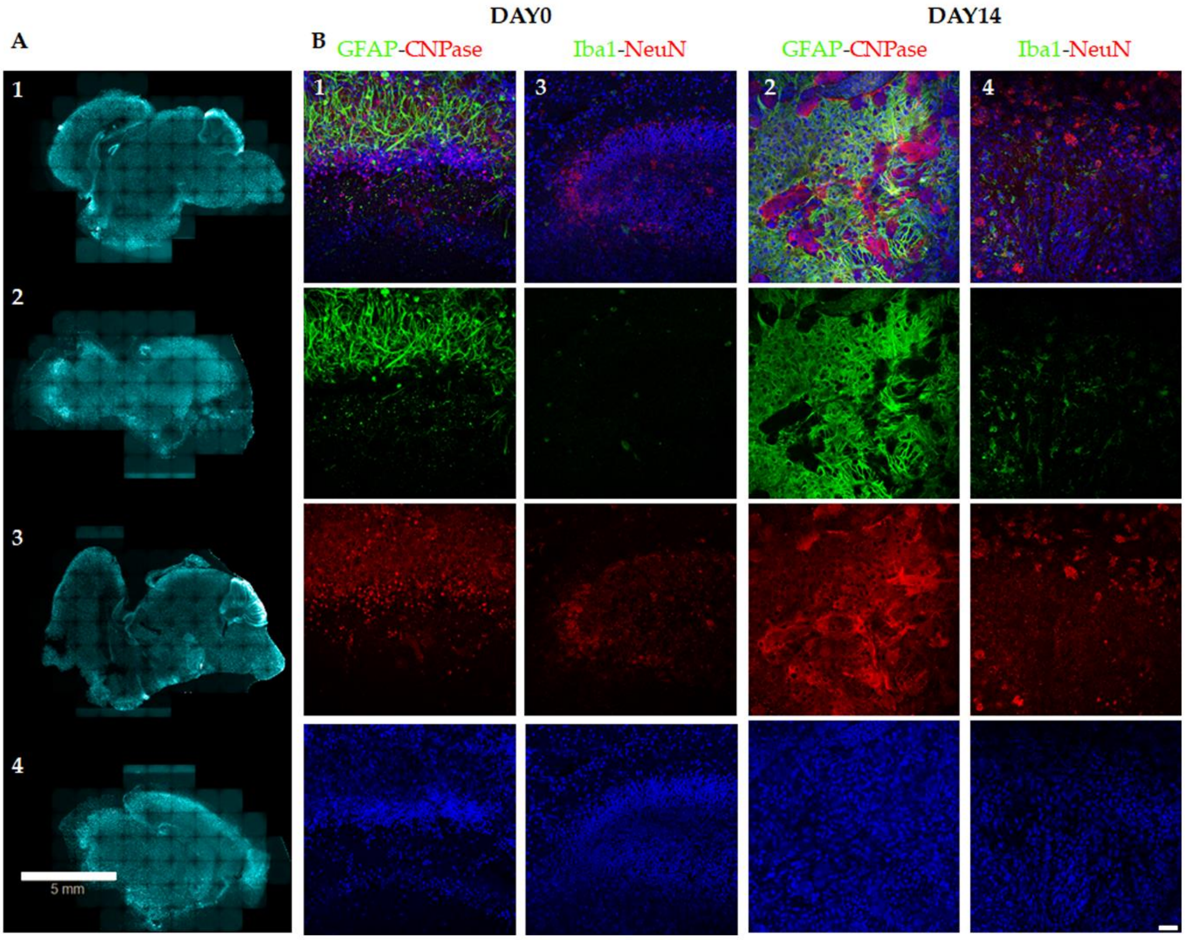

Figure S8. Cytoarchitecture characterization of organotypic brain slices. (**A**) Representative images of sagittal whole organotypic brain slices at day 0 (1 and 3) and two weeks post-preparation (2 and 4) encompassing pons and medulla: Hoechst 33342 (blue). The image was acquired at Operetta CLS in mosaic mode. Bars: 5 mm (**B**) Representative images of organotypic brain slices at day 0 (1 and 3) and two weeks post-preparation (2 and 4), stained for markers of the specific cell types of cerebral tissue: GFAP (green) for astrocytes and CNPase (red) for oligodendrocytes (1 and 2); Iba1 (green) for microglia and NeuN (red) for neurons (3 and 4). The images were acquired at Leica TCS AOBS-SP8X confocal microscope. Bars: 100µm.

Supplementary Videos. 3D migration assay. Representative time lapse of the 3D migration assay for two OPBG-GBM002 individual single cell-derived clones, in mono-culture (**Video S1**, clone 1D3 and **Video S2**, clone 5E2) and in co-culture 1:1 (**Video S3**). Video show the first 15 frames of a total of 96 time points acquired 24 h after the migration was set up. Live fluorescent imaging was performed at the Operetta CLS (PerkinElmer) using the objective 20×. M-orange (yellow) identify the clone 1D3 and Venus (magenta) identify the clone 5E2.
